# Machine Learning-Enabled Meta-Analysis of Bioengineering Micro- and Nanorobots

**DOI:** 10.1021/acs.nanolett.6c02258

**Published:** 2026-09-27

**Authors:** Ehsan Rahimi, Mario Palacios-Corella, Mohammad Rahimi, Lucky Odirile Mohutsiwa, Josep Puigmartí-Luis, Salvador Pané

**Affiliations:** † Faculty of Mechanical Engineering (ME), Delft University of Technology, Mekelweg 2, 2628 CD Delft, The Netherlands; ‡ Departament de Ciència dels Materials i Química Física, Institut de Química Teòrica i Computacional, 16724 Universitat de Barcelona, Barcelona 08028, Spain; § Department of Mechanical Engineering, 3710 McMaster University, Hamilton, ON L8S 4L7, Canada; ∥ Multi-Scale Robotics Lab, Institute of Robotics and Intelligent Systems, Department of Mechanical and Process Engineering, ETH Zurich, Tannenstrasse 3, Zurich 8092, Switzerland; ⊥ Institució Catalana de Recerca i Estudis Avançats (ICREA), Pg. Lluís Companys 23, Barcelona 08010, Spain

**Keywords:** Micro- and nanorobotics, Machine learning, Literature meta-analysis, Bioengineering, Velocity prediction, Actuation classification, Lifetime modeling

## Abstract

Micro- and nanorobots are emerging bioengineering tools for targeted delivery, biosensing, imaging, and minimally invasive therapy, yet systematic design and cross-study translation remain hindered by heterogeneous experimental conditions, inconsistent reporting, and sparse structured data sets. Here, we develop a literature-derived data set of micro- and nanorobot systems and apply artificial intelligence (AI)-enabled structural meta-analysis to resolve relationships among bioengineering constraints, actuation strategies, environmental conditions, measurement practices, and performance. Dendrogram- and correlation-based clustering identifies three dominant descriptor modules spanning deployment context, actuation–measurement coupling, and design intent. Supervised learning reveals structured descriptor patterns in propulsion mechanisms, whereas prediction of instantaneous velocity remains only moderately generalizable, highlighting substantial domain shift across materials, propulsion physics, biological media, operating conditions, and measurement protocols. Overall, AI and machine learning provide a powerful framework for mapping heterogeneous design landscapes, exposing reporting biases and descriptor coupling, and advancing standardized data sets for reliable comparison and predictive design.

## Graphical abstract

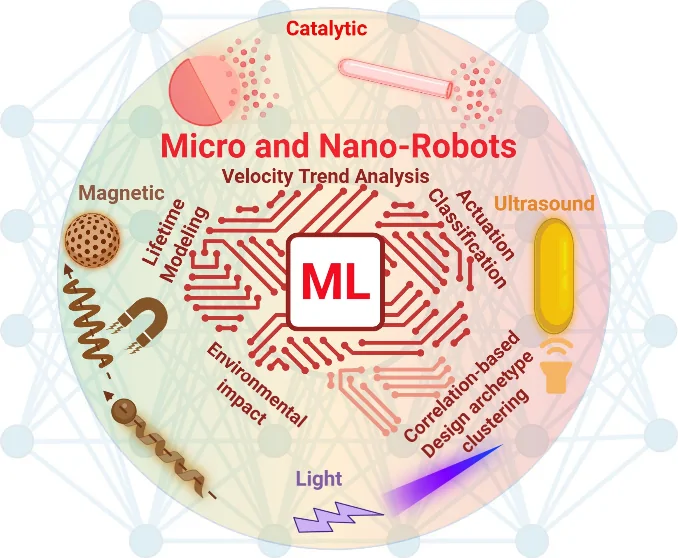

## 1 Introduction

Micro- and nanorobots are untethered, small-scale artificial devices that can be designed and actuated to navigate complex fluids for carrying out specific tasks.^1,2^ Due to their reduced size, they operate at low Reynolds numbers, where viscous forces dominate, and propulsion relies on nonreciprocal motion.^3,4^ This has prompted the exploration of a wide range of actuation strategies, including catalytic/chemical propulsion,^5–8^ magnetic actuation,^9–13^ light-driven systems,^14–16^ ultrasound-based motion,^17–20^ and biohybrid approaches,^21–23^ which, together with diverse geometries, material architectures, and surface functionalization schemes, enable controlled motion and task-specific operation in a variety of environments.^24,25^ Micro- and nanorobots have emerged as promising systems in bioengineering for biomedical scenarios, including targeted drug delivery,^10,26–28^ minimally invasive therapy,^17,22,29–31^ biosensing,^32–36^ diagnostics,^37–39^ and assisted imaging,^40–42^ where they require a controlled operation under complex biological and physicochemical conditions.^9,43,44^ Despite this potential and the substantial progress of the last two decades,^9,24,45^ the micro- and nanorobotics literature is still highly scattered. Original experimental data sets, such as trajectory records or imaging sequences, are rarely shared. Instead, studies more commonly report summary descriptors, including geometry, material composition, propulsion mechanism, environmental or medium conditions, and performance metrics such as velocity or lifetime.^46–48^ As a result, existing work forms a heterogeneous collection of partially overlapping descriptors rather than a harmonized data set, which makes it difficult to trace how design choices, operating environments, and performance outcomes relate across literature. Because most studies are conducted under specific geometries, material systems, and environmental conditions, the resulting performance claims are often localized and difficult to compare or generalize across reports.^2,49^ This heterogeneity naturally raises the question of how bioengineering constraints, experimental context, and design choices co-occur across reported micro- and nanorobot systems and what these patterns imply for data-driven modeling and design.

Machine learning (ML) is well-suited to address these questions and can be used not only for predictive modeling but also for identifying patterns and relationships within heterogeneous data sets.^50–55^ ML can also be used as a structural analysis tool to identify groups of commonly reported variables, measure how often certain descriptor combinations appear, and reveal hidden trends and reporting biases in the literature. However, ML has rarely been used for literature-based meta-analysis in micro- and nanorobotics, and a structured descriptor-based data set is still lacking for this type of analysis.

In this study, we address this gap by constructing a literature-derived data set of 100 micro- and nanorobot systems, each encoded through a consistent set of design, actuation, environmental, and performance descriptors extracted from peer-reviewed studies, as illustrated in Figure 1. Within this framework, we use ML as a structural analysis tool to identify relationships among experimental context, propulsion mechanisms, and bioengineering constraints and to assess how far summary-level literature data can support quantitative performance modeling and design-oriented analysis. Hence, the main contributions of this work are as follows: i. A structured literature-derived data set of 100 micro- and nanorobot systems is developed by organizing reported design, actuation, environmental, and performance descriptors into a unified framework. ii. The study provides a descriptor-based representation of the micro- and nanorobotics literature, enabling systematic comparison across studies with different materials, geometries, propulsion mechanisms, and operating conditions. iii. Correlation-based clustering is used to identify groups of related descriptors and reveal how bioengineering constraints, experimental environments, actuation methods, and measurement strategies co-occur across the field. iv. Supervised ML analysis is applied to evaluate the extent to which summary-level literature descriptors can support classification and performance prediction, including propulsion mechanism classification and velocity prediction. v. The work highlights the limitations of current literature-level data for predictive modeling and provides a data-driven framework to guide more consistent reporting, future data set construction, and design-oriented analysis in bioengineering micro- and nanorobotics.

**Figure 1:**
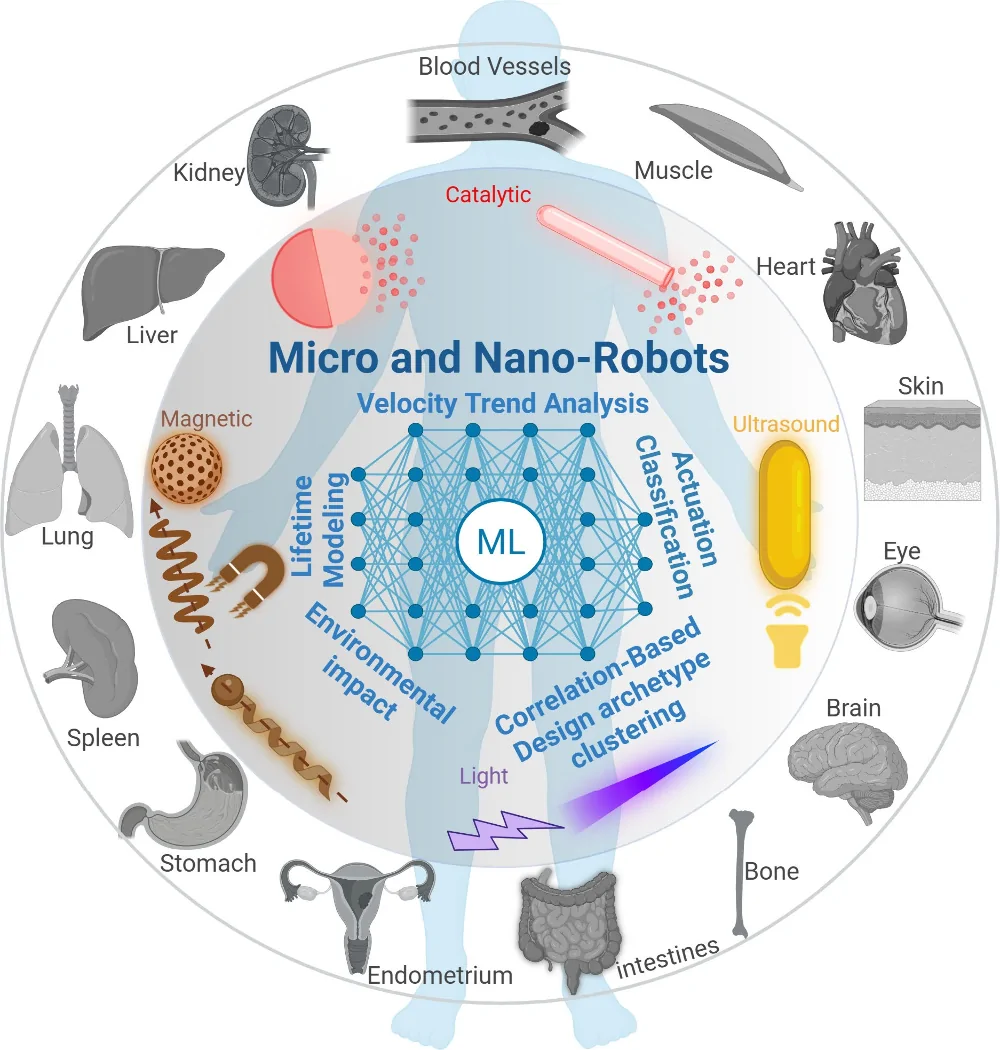
Schematic representation of an ML approach for bioengineering-oriented micro- and nanorobots, in which literature-reported design, actuation, and environmental parameters are structured into ML-interpretable features and linked to multiobjectives, including velocity trend analysis, actuation mechanism classification, lifetime and degradability modeling, and unsupervised correlation clustering for design- and context-level pattern discovery. ML is used as a data-driven analytical layer for cross-study meta-analysis rather than real-time robotic control. Created in BioRender. Rahimi, M. (2026) https://BioRender.com/2eyf3g0.

## 2 Descriptors for Micro- And Nanorobots

Prior to the ML-based analysis, the design landscape of bioengineering-oriented micro- and nanorobots should be defined, as the selection and organization of descriptors strongly influence the trends, correlations, and structural patterns captured from the literature-derived data set. The design space of bioengineering-oriented micro- and nanorobot systems encompasses the variables that define how these systems are constructed, operated, and evaluated within biological and physicochemical environments. The details of the data set and features used in this study are provided in Table S1 of the Supporting Information (SI). These variables include geometry, material composition, propulsion mechanism, functionalization, environmental conditions, biological medium, and performance metrics, which collectively determine system behaviors and applicability as illustrated in Figure 2.^1,48^ However, this design space is inherently hierarchical as not all variables contribute equally to system feasibility and performance. In practice, micro- and nanorobot design is first constrained by bioengineering requirements, which define the feasible operating domains. Within these constraints, design and actuation choices determine system behavior, while additional modifiers and environmental conditions influence the performance and its interpretation. Accordingly, the design space is organized into four interacting categories: **a**. bioengineering constraints, **b**. design and actuation variables, **c**. secondary modifiers and functionalization, and **d**. environmental and measurement context. This hierarchy provides a structured representation of the design space, where constraints define feasibility, design variables determine function, modifiers refine behavior, and the environmental context governs performance outcomes. This organization forms the basis for the subsequent ML analysis, where these descriptors are used to identify structural relationships and co-occurrence patterns across the studies. Bioengineering constraints represent the most fundamental layer, defining the feasible operating domain of biomedical applications. These include biocompatibility, degradability, immune response, clearance pathways, and compatibility with biological media, as shown in Figure 2c and d. For example, systems intended for *in vivo* applications must avoid toxic materials and ensure safe degradation, while operation in biological fluids imposes constraints on viscosity, ionic strength, and chemical composition.^45,56–59^ These constraints directly limit the range of viable geometries, materials, and propulsion strategies.

**Figure 2:**
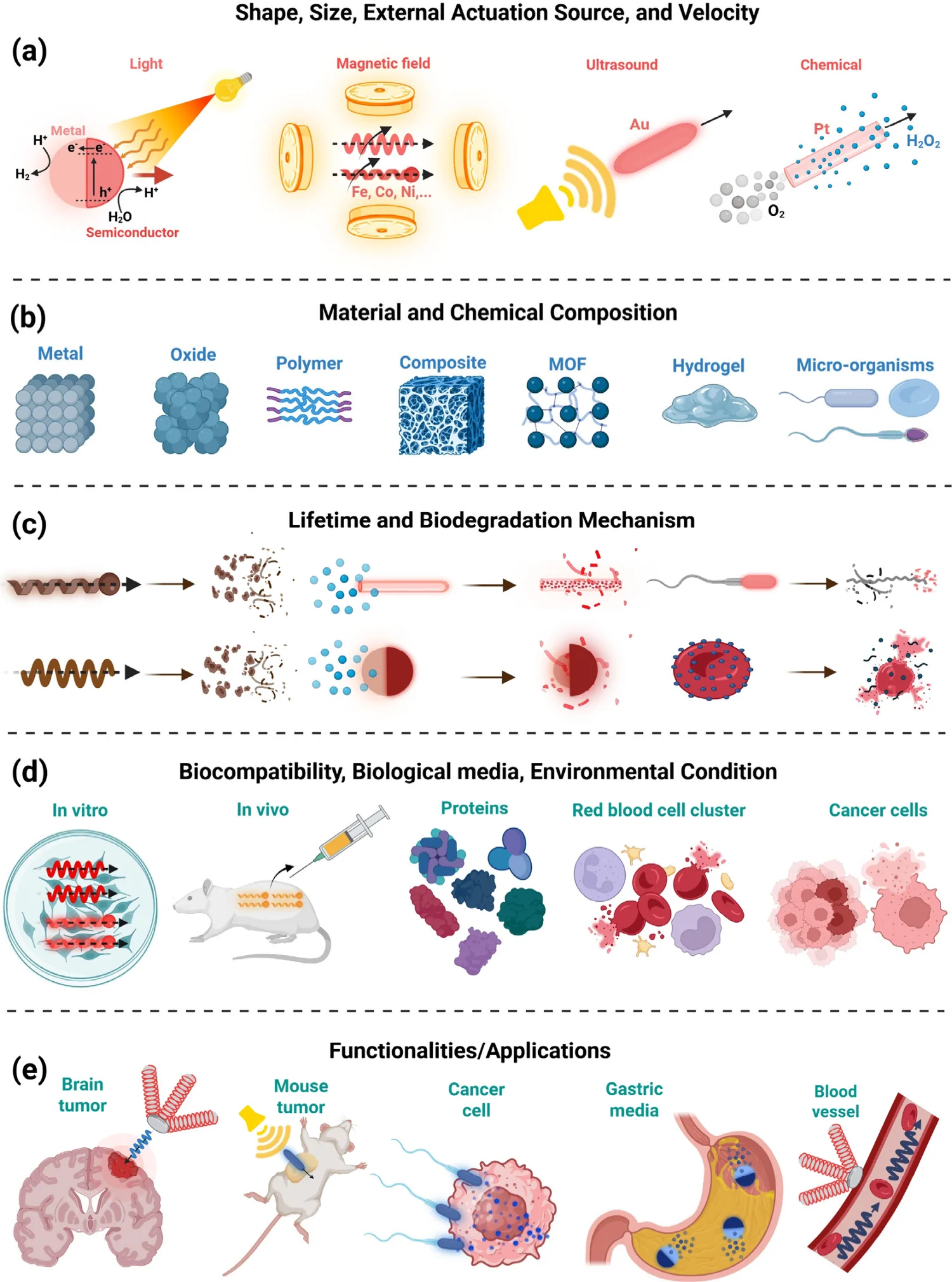
Schematic representation of micro- and nanorobots with multidimensional design space, illustrating (a) geometries and actuation modes, (b) material classes and multifunctional integrations, (c) bioengineering constraints including lifetime and biodegradation mechanism, (d) the influence of biological media, biocompatibility, and environmental conditions on propulsion and stability, and (e) various applications and functionalities of micro- and nanorobots in biomedical. All these factors have been considered in an ML framework for bioengineering-oriented micro- and nanorobots. Created in BioRender. Rahimi, M. (2026) https://BioRender.com/2eyf3g0.

Within these constraints, design and actuation variables determine the physical behavior of micro- and nanorobotic systems. Key design variables include geometry, material, and chemical composition, encompassing metals,^14,60–62^ polymers,^17,63,64^ hydrogels,^17,63,64^ and biodegradable composites,^65–67^ while propulsion strategies define how these systems convert external or locally generated energy into controlled motion, as illustrated in Figure 2a and b. At low Reynolds numbers, where viscous forces dominate over inertia, propulsion generally relies on nonreciprocal motion or asymmetric physicochemical interactions with the surrounding medium.^54^ Among the principal actuation strategies, catalytic/chemical propulsion generates motion through localized chemical reactions, concentration gradients, or gas evolution and can provide autonomous propulsion without continuous external fields.^48,68^ However, its biomedical applicability may be constrained by fuel requirements, reaction byproducts, and sensitivity to local chemical conditions.^3^ Magnetic actuation instead enables wireless and highly controllable propulsion through externally applied magnetic fields and is particularly attractive for biomedical applications because magnetic fields can penetrate biological tissues while permitting directional steering and trajectory control.^69,70^ Light-driven systems exploit photothermal, photochemical, or photocatalytic effects to achieve remotely regulated motion with a high spatial and temporal precision. However, their performance in vivo can be limited by light penetration and scattering in deeper tissues.^16,33^ Acoustic or ultrasound-driven actuation provides another noncontact approach, using acoustic forces, streaming, or oscillatory phenomena to generate propulsion and manipulate micro- and nanoscale systems.^71,72^ Its ability to penetrate relatively deep into tissue and its compatibility with ultrasound imaging have motivated recent demonstrations of helical propulsion, microbubble-assisted transport, soft actuation, and real-time microrobot tracking.^73–76^ More recently, model-based reinforcement learning has also been integrated with ultrasound-driven systems to support adaptive navigation under complex vascular and flow conditions.^77^ Collectively, these actuation strategies present different trade-offs in propulsion efficiency, controllability, penetration depth, biocompatibility, and dependence on the surrounding environment, making propulsion selection closely coupled to the intended biomedical application and deployment conditions. Beyond primary design variables, secondary modifiers and functionalization strategies further refine system behavior by enabling application-specific capabilities. These include surface coatings, targeting ligands, responsive polymers, and payload integration for delivery or sensing applications.^78,79^ While they do not define the primary actuation mechanism, they are critical for controlling interactions with biological environments, improving selectivity, and enhancing stability and functionality.^48^

Finally, the environmental and measurement context govern both system performance and the interpretation of reported results. As illustrated in Figure 2d, variables such as pH, temperature, medium composition, and deployment context (in vitro or in/ex vivo) influence propulsion efficiency, reaction kinetics, and biological interactions.^56,58,80^ In addition, performance metrics such as velocity and lifetime depend on measurement conditions and reporting practices, making them essential descriptors for cross-study comparisons. Practical deployment is further constrained by limitations in localization and tracking, as conventional positioning systems are not applicable at micro- and nanoscales and clinical imaging modalities often provide limited resolution, as illustrated in Figure 2e.^81^ These categories define a structured descriptor framework that captures both engineering and bioengineering dimensions of micro- and nanorobotic systems. This framework enables systematic analysis of how design variables, experimental contexts, and performance metrics co-occur across the literature and provides the foundation for the ML-based meta-analysis presented in the following sections.

## 3 Machine Learning Framework

Data set construction followed a structured search of peer-reviewed studies published between 2010 and 2026. Eligible records described a distinct bioengineering-relevant micro- or nanorobot system and reported sufficient system-level information to extract, at minimum, the propulsion mechanism together with design/material and experimental context descriptors. Reviews, duplicate systems, purely theoretical or control studies without extractable system-level data, and reports lacking the minimum descriptor set were excluded. The resulting collection should therefore be interpreted as a broad descriptor-coverage sample rather than an exhaustive census of the field; full search and screening details are provided in Section S1.1 and S1.2 of the SI.

To enable computational analysis, the extracted descriptors were encoded into ML-interpretable features as illustrated in Figure 3. Categorical variables such as propulsion mechanism, material class, biological medium, and application type were transformed using one-hot encoding, where each category was represented as a separate binary indicator variable. This avoids imposing artificial ordinal relationships among nominal descriptors such as magnetic, catalytic, acoustic, or light-driven propulsion. The propulsion mechanisms, such as magnetic, catalytic, acoustic, and light-driven actuation, were encoded as separate binary features indicating the presence or absence of each mechanism in each system. Continuous variables, including instantaneous velocity, temperature, and pH, were kept in their original reported numerical format. The resulting feature space was then organized into physically meaningful groups, such as shape, material, propulsion, environment, and measurement descriptors, to define supervised and unsupervised ML tasks. Specifically, regression tasks were used to evaluate relationships between design, environmental conditions, and performance metrics, including velocity prediction and lifetime or degradation modeling. Classification was applied to propulsion mechanisms to assess the internal consistency and discriminative power of literature-derived descriptors. In addition, unsupervised analysis and clustering were used to identify co-occurrence patterns and potential archetypes of micro- and nanorobot systems. These task definitions reflect the coupling between design variables, experimental context, and performance outcomes in micro- and nanorobot studies. Dendrogram correlation-based clustering (DCC) was used to analyze relationships among descriptors across the data set.^82,83^ In this approach, Pearson correlations between variables are computed and used to construct a similarity matrix, which is then organized using hierarchical clustering to identify groups of covarying descriptors. This method does not assume causal relationships but instead captures statistical co-occurrence patterns among variables reported in the literature. The resulting clustered correlation matrix provides a compact representation of descriptor relationships across heterogeneous studies. Besides, correlation coefficients range from −1 to +1, where positive values indicate systematic coreporting or alignment of descriptors, and negative values reflect mutually exclusive or inversely associated experimental regimes. DCC was applied to evaluate the multivariate relationships between descriptors and outputs. As the first step, all variables are standardized to a zero mean and unit variance. Next, a Pearson correlation analysis was applied by computing the pairwise correlation coefficients ρ_*ij*_ for all variable pairs, and a correlation-derived distance metric was subsequently defined (eq 1):

1 $$d_{ij=1‐\left|pij\right|}$$

**Figure 3:**
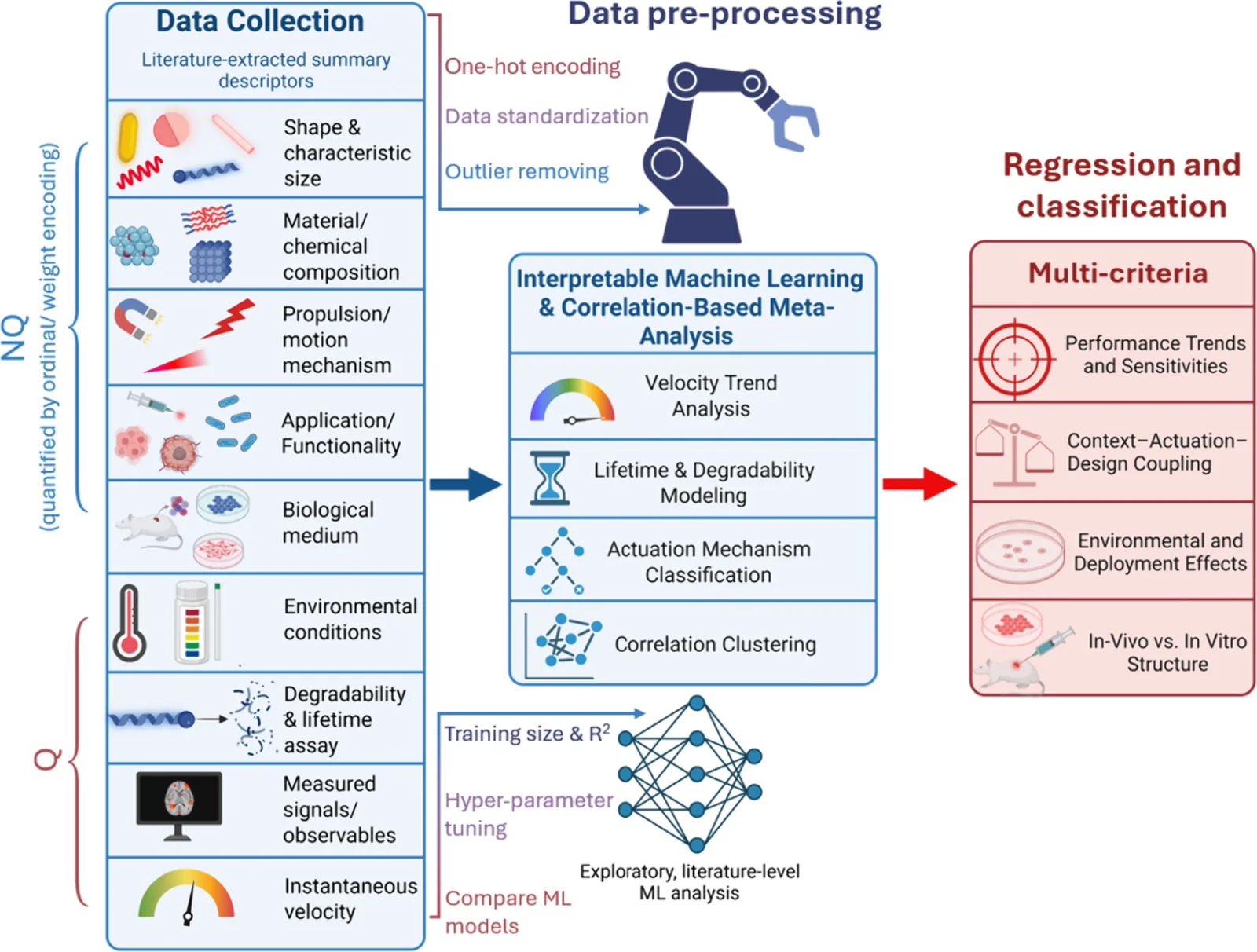
Workflow illustrates a literature-level ML analysis framework, where summary descriptors extracted from published studies are used for correlation clustering and illustrative modeling, rather than for optimization or real-time control. Created in BioRender. Rahimi, M. (2026) https://BioRender.com/2eyf3g0.

DCC, therefore, identifies groups of descriptors that covary or show structured relationships with outputs. These clusters can be interpreted as distinct regions of a design landscape, where (i) bioengineering constraints define feasible operating regimes, (ii) actuation and measurement strategies coevolve, and (iii) material and functional choices reflect application-specific requirements. In addition to unsupervised analysis, supervised learning tasks were defined to evaluate the extent to which literature-derived descriptors contain predictive information about the selected outcomes. These tasks include classification of propulsion or motion mechanisms and regression of performance-related variables such as instantaneous velocity and degradability or lifetime metrics. Representative models were selected to capture both nonlinear and baseline trends: gradient boosting regression, XGBoost, extremely randomized trees, Ridge, and ElasticNet for regression and random forest and support vector machine models for propulsion-mechanism classification. These models were selected because they are suitable for small, heterogeneous tabular data sets while balancing nonlinear learning capacity and interpretability. More complex deep-learning models were not prioritized due to limited data set size, uneven descriptor distributions, and increased overfitting risk, supporting the view that data heterogeneity rather than model capacity is the main limitation. Standard ML models were trained and evaluated using appropriate data splits, and feature importance was assessed by using SHAP (Shapley Additive Explanations) to quantify the contribution of individual descriptors to model predictions. Given the modest data set size and heterogeneous literature-derived nature of the data, model evaluation was performed with particular emphasis on robustness and overfitting control rather than benchmark-level prediction. The data set was divided into training and testing subsets, and hyperparameter optimization was conducted using cross-validation within the training set to avoid information leakage. Model performance was then evaluated on the held-out test set, while the consistency between training and testing scores was used to assess the potential overfitting. Because the data set contains approximately 100 systems and uneven class/descriptor distributions, the supervised models are interpreted as illustrative tools for evaluating descriptor consistency and predictive feasibility rather than definitive predictive models. The optimized hyperparameters of all ML models, obtained through cross-validated search procedures, are summarized in the Supporting Information (SI), Table S2.

## 4 Results and Discussion

### 4.1 Descriptor Relationships Revealed by DCC Analysis

The application of the DCC^82–84^ to the literature-derived data set reveals three dominant descriptor clusters, as illustrated in Figure 4. The first cluster corresponds to the experimental context and deployment domain, including variables such as in vitro or in/ex vivo conditions, temperature, and degradability or lifetime assays. The second cluster captures the actuation strategy and measurement practice, linking the propulsion or motion mechanism with measured signals or observables and instantaneous velocity.

**Figure 4:**
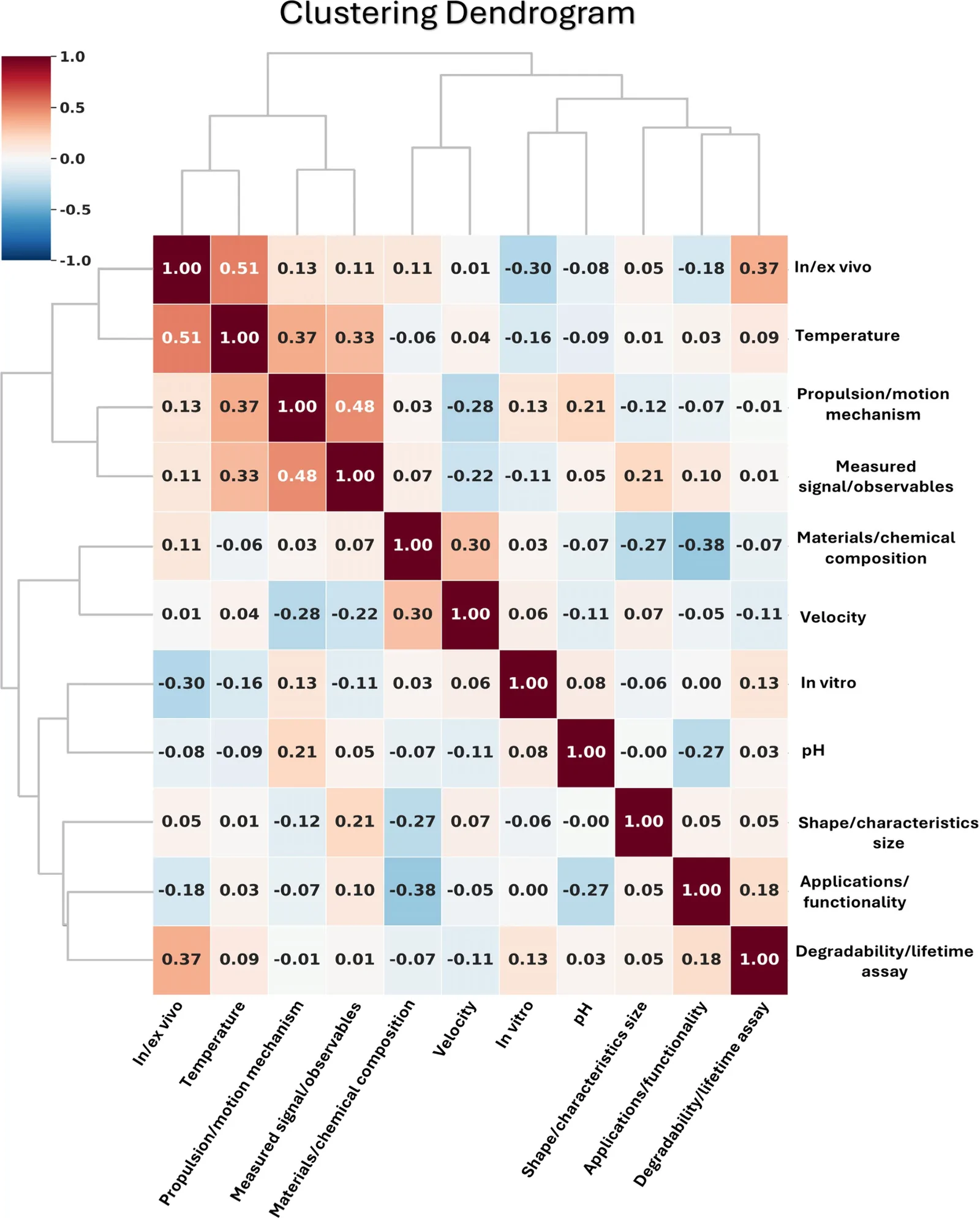
Clustered Pearson correlation heatmap with hierarchical dendrograms showing relationships among experimental context, design descriptors, environmental conditions, and performance metrics in micro- and nanorobot studies. Three main clusters are identified: deployment context, actuation/measurement practice, and design intent under bioengineering constraints. Correlation values are shown in each cell, with colors ranging from −1 to +1. This unsupervised analysis highlights co-occurrence patterns and potential confounding effects, rather than causal relationships, emphasizing the influence of domain-dependent experimental context on ML performance.

The third cluster reflects design intent under bioengineering constraints, including material composition, shape, and characteristic size, application or functionality, and environmental variables, such as pH and temperature. These clusters indicate that reported micro- and nanorobot performance does not arise from isolated design parameters but from coordinated combinations of environment, actuation, sensing, and application focus. Importantly, the clustering also reflects how the community reports systems reveal consistent co-occurrence patterns such as the association between biological deployment contexts and degradability assessment and the coupling between propulsion mechanisms and measurement modalities. This suggests that the observed structure captures both physical relationships and systematic reporting practices in the literature. Based on this structured organization of descriptors, subsequent ML analyses are defined to probe different aspects of the data set, including regression tasks for performance metrics (e.g., velocity and lifetime), classification of propulsion mechanisms, and unsupervised grouping of system archetypes. These tasks provide complementary perspectives on how design variables, experimental contexts, and performance outcomes are coupled across studies.

#### 4.1.1 Experimental Context, Deployment Domain, And Stability Emphasis (Cluster 1)

Cluster 1 captures the experimental framing and translational intent underlying reported micro- and nanorobot studies rather than intrinsic design or actuation physics. The strongest correlation is observed between the in/ex vivo context and temperature (r = 0.51), indicating that studies in biologically relevant environments are typically conducted under physiologically constrained thermal conditions. In contrast, in vitro studies allow for broader thermal variation, reflecting fewer biological constraints. As encoded in this data set, in/ex vivo and in vitro variables exhibit a negative correlation (r = −0.30), capturing the dominant experimental context assigned to each system rather than implying strict exclusivity across studies. The emergence of this structure confirms the internal consistency of the data set and highlights the systematic reporting of experimental context in the literature.^85–88^ A key observation is the positive correlation between the in/ex vivo context and degradability and lifetime assays (r = 0.37), indicating that stability and degradation are more frequently evaluated in biologically relevant studies. In contrast, many in vitro investigations prioritize motion and control without assessing the long-term material behavior. This pattern reveals a reporting bias driven by application intent, where stability becomes a priority primarily in translational settings. From an ML perspective, this coupling implies that performance metrics are not independent of the experimental context. Consequently, models trained on literature-derived data must account for the systematic underrepresentation of lifetime-related descriptors in in vitro studies as ignoring this imbalance may lead to misleading interpretations. This reinforces the need for context-aware features and highlights experimental context as a key source of domain shift in bioengineering-oriented micro- and nanorobotics.

#### 4.1.2 Actuation Strategy and Measurement Coupling (Cluster 2)

Cluster 2 represents the functional core of micro- and nanorobot operations, linking actuation mechanisms, measurement strategies, and performance quantification. The strongest association is observed between the propulsion mechanism and measured signals/observables (r = 0.48), indicating that actuation and tracking modalities are tightly coupled. For example, magnetic actuation is typically paired with trajectory tracking under externally applied fields, while chemically propelled systems are commonly observed via optical microscopy in reactive environments, and photo- or acoustically driven systems rely on modality-specific imaging approaches. This coupling reflects not only the physical compatibility but also experimental practicality. Magnetically actuated systems enable precise, reversible control, facilitating reliable tracking, whereas chemically propelled systems often exhibit stochastic motion, and acoustic or optical actuation remains less standardized in terms of instrumentation and control. As a result, different actuation strategies are associated with distinct levels of control and measurement consistency. The moderate correlation between the propulsion mechanism and temperature (r = 0.37) further indicates that actuation strategies are explored under different thermal regimes, with chemically driven systems often requiring controlled conditions to sustain reaction kinetics, while magnetic systems are more commonly studied under near-physiological environments. A secondary observation is that instantaneous velocity shows weak correlations with propulsion mechanism with r of −0.28 and measured signals with value of r = −0.22, indicating that actuation category alone cannot explain velocity trends across studies. Instead, velocity reflects coupled effects of material composition, surface chemistry, and environmental conditions with significant variability within each propulsion class. This is supported by its positive correlation with the material/chemical composition (r = 0.30), highlighting the influence of catalytic activity, magnetic properties, or surface functionalization. More importantly, this weak correlation structure reveals that velocity is highly context-dependent and sensitive to experimental conditions, including medium composition and the physicochemical environment. Consequently, similar systems can exhibit markedly different velocities across studies, introducing a domain shift that limits the ability of ML models to capture consistent predictive relationships. From a bioengineering perspective, velocity should therefore be interpreted as an emergent system-level response rather than a primary design variable and is treated here as a secondary, illustrative metric.

#### 4.1.3 Design Intent, Application Constraints, and Environmental Compatibility (Cluster 3)

Cluster 3 encapsulates design intent and bioengineering constraints, revealing how material choices, geometry, and operating conditions align with targeted applications. One of the strongest signals in this cluster is the negative correlation between material/chemical composition and application/functionality which r was achieved −0.38. This suggests that materials are not selected uniformly across applications; rather, certain compositions are ideal or avoided depending on functional goals such as therapy, imaging, or sensing. For example, highly reactive or toxic materials may be acceptable in short-lived in vitro demonstrations but are systematically excluded from biologically oriented applications. The negative correlation between pH and application/functionality with r = −0.27, further supports this interpretation. Extreme pH environments are predominantly associated with catalytic propulsion studies or proof of concept demonstrations, whereas biologically relevant applications are usually tested near neutral pH. This separation highlights the role of environmental compatibility as an implicit design constraint, encoded indirectly through reported experimental conditions. Degradability and lifetime show positive correlations with application/functionality (r = 0.18) and in/ex vivo context (r = 0.37), indicating that stability and controlled degradation become more prominent as systems move toward translational applications. Although the correlation with application is modest, its consistent direction suggests a systematic shift in design priorities driven by bioengineering requirements. In contrast, shape and characteristic size exhibit weak correlations (|r| ≈ 0.05 to 0.21), indicating that geometric diversity persists across applications and environments. This suggests that geometry remains a flexible design parameter, whereas material composition and environmental compatibility impose stronger constraints, with geometry contributing primarily to intracluster variability rather than high-level design structure. Together, the three clusters reveal a layered structure in the micro- and nanorobotics literature. Cluster 1 defines experimental and translational context, Cluster 2 captures motion implementation and tracking, and Cluster 3 reflects design intent under bioengineering constraints. These partially coupled layers generate structured correlations that ML can use for exploratory analysis and trade-off visualization. Thus, DCC positions ML not as a predictive oracle, but as a structural tool to reveal how experimental choices, design decisions, and performance metrics coevolve across the field.

### 4.2 Supervised Learning Analysis of Propulsion, Velocity, and Lifetime

The supervised learning framework was used to evaluate whether literature-derived descriptors contain sufficient information to discriminate propulsion mechanisms and model performance-related variables, including instantaneous velocity and degradability and lifetime criteria. These tasks complement the DCC analysis by quantitatively assessing descriptor consistency, predictive feasibility, and limitations arising from data set heterogeneity. Figure 5 summarizes the supervised learning results. For propulsion-mechanism classification (Figure 5a), the actual-versus-predicted display is retained only as an auxiliary diagnostic of agreement between encoded labels; because the target is categorical, R^2^ is not treated as a formal classification metric. From the held-out confusion matrix (as can be seen in Figure 6; n = 18), the model achieved an overall accuracy of 76.19%, balanced accuracy (66.14%), and weighted F1 (72.36%). The difference between overall and balanced metrics reflects the strong class imbalance and the limited representation of the minority propulsion classes. Magnetic systems were classified most reliably, whereas errors among the remaining classes indicated overlapping descriptor profiles and insufficient minority-class coverage. Accordingly, classification performance is interpreted primarily from the confusion matrix and class-based metrics, while the R^2^-style plot is used only as a diagnostic visualization. Table 1 is dominated by magnetic and catalytic/chemical systems, whereas light-driven, ultrasound-driven, and unassigned systems are sparsely represented. This imbalance explains why the overall accuracy is substantially higher than the balanced accuracy and limits reliable class-wise generalization.

**Table 1:** Distribution of the Curated Systems by Propulsion Mechanism

Propulsion mechanism	Number of systems	Share of curated table (%)
Magnetic	45	45
Catalytic/chemical	40	40
Light driven	8	8
Ultrasound driven	5	5
Unassigned/NA	2	2.0
Total	100	100

**Figure 5:**
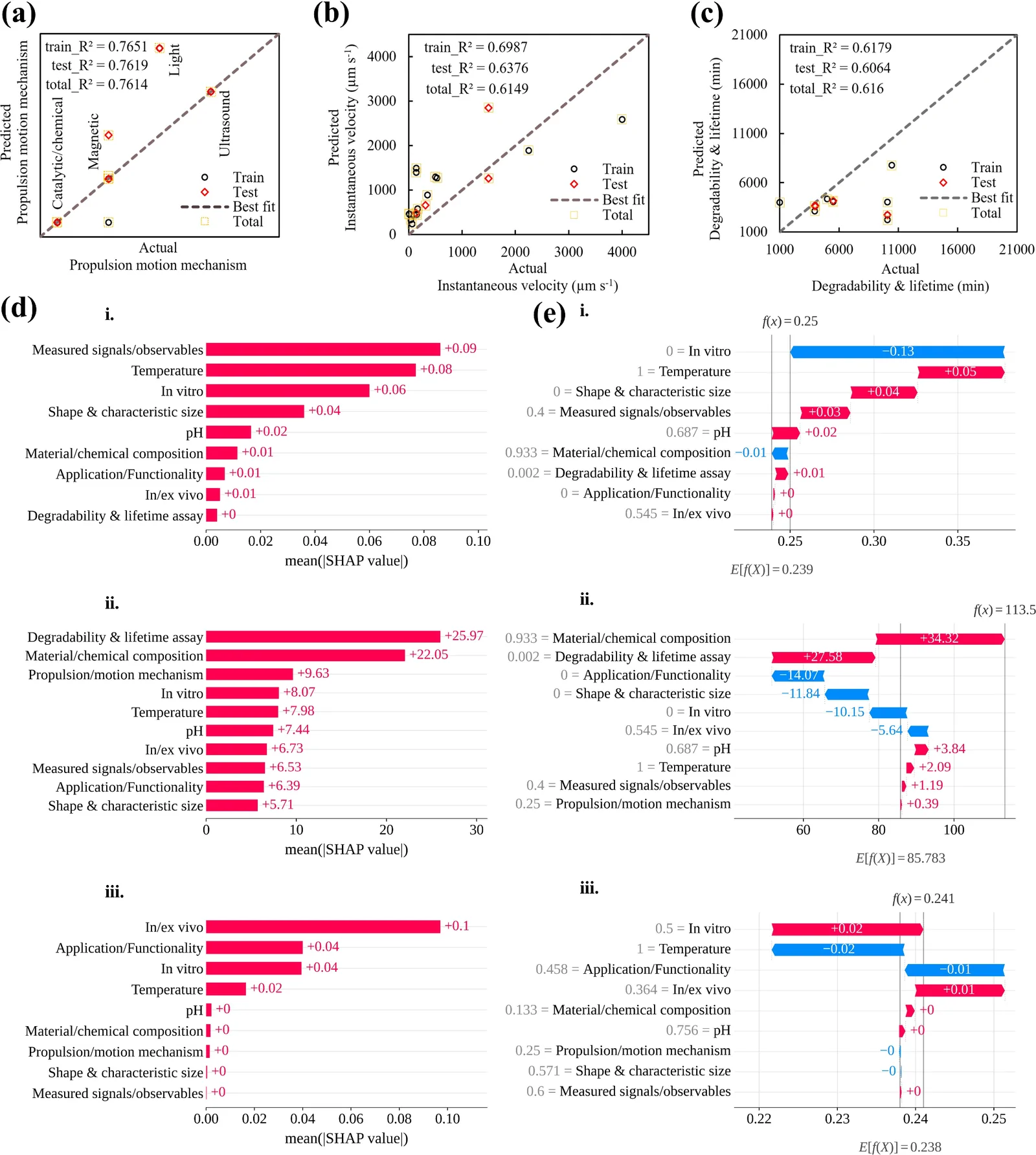
(a) Diagnostic actual versus predicted plot for propulsion-mechanism classification. The dashed line represents ideal agreement. Because propulsion mechanism is a categorical variable, this visualization is used only as a qualitative indicator of global agreement rather than a formal classification metric. (b) Regression results for instantaneous velocity, showing moderate agreement between predicted and actual values with noticeable dispersion, reflecting context-dependent variability. (c) Regression results for degradability and lifetime, indicating partial predictability and consistent training/testing behavior. (d) SHAP (Shapley Additive Explanations) feature-importance analysis: (i) propulsion classification, (ii) velocity prediction, and (iii) lifetime prediction, highlighting the relative contribution of descriptors across tasks. (e) SHAP contribution plots illustrate the direction and magnitude of individual descriptor effects on model outputs for (i) propulsion classification, (ii) velocity prediction, and (iii) lifetime prediction. Overall, the results demonstrate that literature-derived descriptors capture meaningful structural relationships, while predictive performance is limited by data heterogeneity and domain shift across experimental conditions.

**Figure 6:**
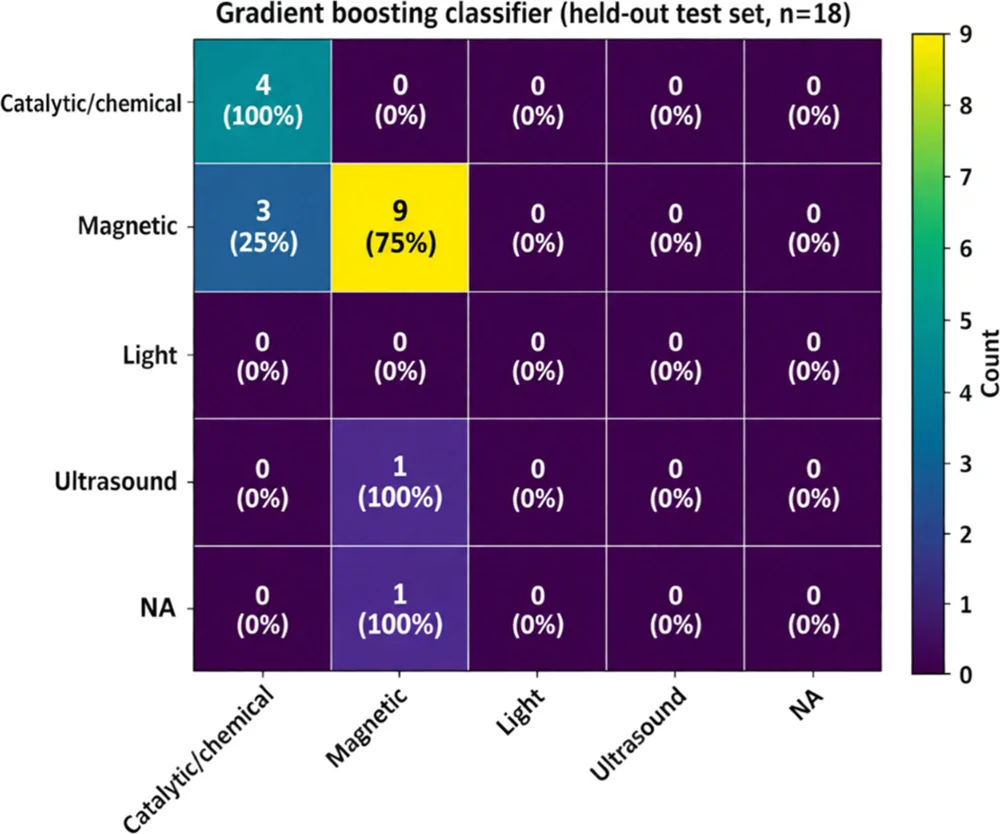
Count and row-normalized confusion matrix for the held-out propulsion-mechanism classification set (*n* = 18). Rows denote true classes, and columns denote predicted classes. Each cell reports the sample count and, where applicable, the percentage within the true class. The strong magnetic diagonal and weaker minority-class performance demonstrate both descriptor consistency for the dominant class and the effect of class imbalance.

Regression analysis of instantaneous velocity (Figure 5b) yields moderate predictive performance (train R^2^ ≈ 0.70, test R^2^ ≈ 0.64), with noticeable dispersion around the parity line. The reduced testing performance and spread of residuals indicate limited generalization, reflecting the strong context dependence of velocity. Variations in medium composition, temperature, and physicochemical environment introduce substantial domain shift, limiting predictive consistency across studies. Consequently, velocity behaves as an emergent system-level response rather than a directly predictable descriptor. A similar trend is observed for degradability and lifetime prediction (Figure 5c), where moderate agreement (train R^2^ ≈ 0.62, test R^2^ ≈ 0.61) suggests partial predictability but persistent variability. The relatively small train-test gap indicates that the limitation arises primarily from intrinsic data heterogeneity and incomplete descriptor representation rather than severe overfitting. The limited velocity performance should not be attributed to domain shift alone. One-hot encoding preserves the identity of an actuation category but does not resolve its operating intensity or underlying propulsion physics. Key continuous parameters, such as magnetic-field amplitude, gradient and frequency; acoustic frequency, pressure and duty cycle; light wavelength and intensity; catalytic fuel concentration; medium viscosity; and confinement, are absent or inconsistently reported. Consequently, systems with substantially different force-generation and transport regimes may receive similar encoded representations, contributing directly to unexplained velocity variance. Robustness and uncertainty analysis of the propulsion classification, velocity regression, and lifetime regression models are provided in Table S3. Feature-importance analysis using SHAP (Figure 5d) provides further insight into descriptor contributions. For propulsion classification (Figure 5d-i), measured signals/observables (∼0.09 mean |SHAP|), temperature (∼0.08), and in vitro conditions (∼0.06) dominate the feature space, indicating that actuation mechanisms are closely linked to observation methods and testing environments. In velocity prediction (Figure 5d-ii), degradability/lifetime (∼25.97), material composition (∼22.05), and propulsion mechanism (∼9.63) are the dominant contributors, highlighting strong coupling between material properties and dynamic performance. For lifetime prediction (Figure 5d-iii), in/ex vivo context (∼0.10) emerges as the most influential descriptor, confirming that degradation trend is strongly governed by deployment conditions. The SHAP contribution plots (Figure 5e) further illustrate the directionality and magnitude of descriptor effects. In velocity prediction (Figure 5e-ii), material composition and degradation-related descriptors introduce strong positive contributions, whereas application and geometric factors produce negative shifts, reflecting competing design constraints. Overall, the supervised learning results demonstrate that literature-derived descriptors encode statistically meaningful structure and enable moderate predictive performance. However, the limited regression accuracy (R^2^ ≈ 0.60–0.65) and observed class overlap indicate that model performance is constrained primarily by data heterogeneity, descriptor coupling, and domain shift rather than model capacity. These findings reinforce that ML currently functions most effectively as a structural and exploratory analysis tool in micro- and nanorobotics when applied to summary-level literature data.

## 5 Limitations

The ML analyses presented in the preceding sections reveal several limitations inherent to the literature-derived data set used in this work, which directly affect the interpretation of both the DCC clustering and supervised learning results. A primary limitation is the scarcity and fragmentation of available data (Figure 7a). The data set comprises 100 systems, with descriptors unevenly distributed across actuation strategies, materials, and experimental contexts. As observed in the DCC analysis, many variables are reported only within specific study types, limiting statistical coverage and increasing the risk of data set bias. This fragmentation constrains the robustness of the correlation patterns and contributes to the moderate performance observed in supervised tasks. A second limitation arises from context-dependent reporting and domain shifts between experimental regimes (Figure 7b). The DCC results show that environmental variables cluster strongly with the deployment context and application intent, indicating that experimental conditions are not independently sampled. As a result, models trained under simplified in vitro conditions may not generalize to biologically relevant environments, where factors such as pH, viscosity, and biochemical composition differ significantly. This coupling must be considered when interpreting both the clustering structures and predictive performance. A further challenge is the presence of label uncertainty in literature-derived descriptors (Figure 7c). Many target variables, including velocity and lifetime, are extracted indirectly from imaging or experimental proxies and may be inconsistently defined across studies. This contributes to the weak correlations observed for velocity in the DCC analysis and limits the accuracy of regression models, reinforcing the idea that summary-level descriptors are insufficient for precise quantitative prediction. Finally, the limited size and heterogeneity of the data set restrict the applicability of complex ML models (Figure 7d). In this context, interpretable approaches such as correlation clustering and simple supervised models are more appropriate as they provide structural insights while remaining consistent with the available data scale. Overall, these limitations indicate that the results presented here should be interpreted as identifying structural trends and reporting patterns rather than as definitive predictive relationships. To improve future interoperability and reduce the reporting gaps identified here, Table 2 summarizes a minimum reporting checklist for bioengineering-oriented micro- and nanorobot studies.

**Figure 7:**
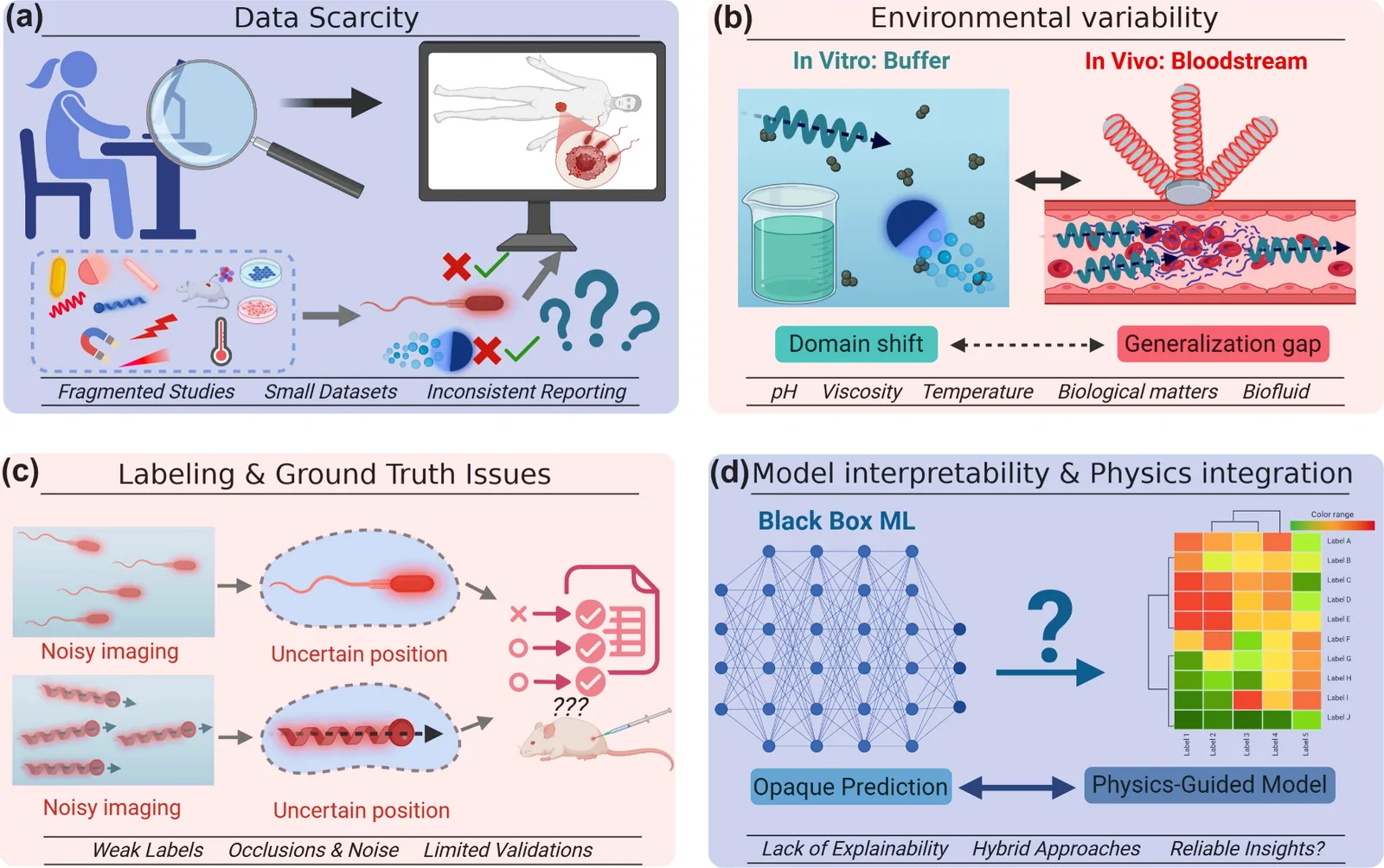
Key challenges for ML in micro- and nanorobotics, including (a) data scarcity and fragmentation, (b) environmental variability and domain shift, (c) labeling and ground-truth uncertainty, and (d) limited interpretability and physics integration. These challenges highlight the need for standardized reporting, environment-aware models, and interpretable physics-guided ML for reliable bioengineering translation. Created in BioRender. Rahimi, M. (2026) https://BioRender.com/2eyf3g0.

**Table 2:** Recommended Minimum Reporting Checklist for Future Micro- and Nanorobot Studies

Category	Minimum information to report
System design	Shape, characteristic dimensions, size distribution, fabrication route, and batch/sample number
Materials	Full composition, loading or layer thickness, surface coating/functionalization, payload, and relevant physicochemical properties
Operating medium	Complete composition, ionic strength, viscosity, additives/fuels, flow rate, and confinement geometry
Environmental and hydrodynamic conditions	Static or flowing condition, flow velocity/shear rate, confinement geometry, oxygenation level where relevant, and applied pressure where relevant
Actuation parameters	Modality-specific intensity: magnetic field/gradient/frequency; acoustic frequency/pressure/duty cycle; light wavelength/intensity; catalytic fuel concentration
Tracking and measurement	Imaging modality, spatial/temporal resolution, frame rate, tracking algorithm, calibration, and uncertainty
Velocity and motion	Definition of instantaneous/mean/peak velocity, analysis window, number of trajectories, sample size, variability, and statistical uncertainty
Lifetime/degradation	Assay definition, end point, medium and conditions, observation duration, degradation products, and uncertainty
Data availability	Raw trajectories/images, processed data, code, and machine-readable metadata or repository identifier

## 6 Conclusions and Future Perspectives

In this work, we present an ML-enabled framework for literature-level meta-analysis of micro- and nanorobots based on a curated data set of ∼ 100 systems. By transforming heterogeneous experimental reports into a unified descriptor-based representation, we show that summary-level variables are sufficient to uncover statistically consistent structural relationships across studies. DCC analysis reveals three dominant descriptor clusters: deployment context, actuation and measurement practice, and bioengineering-driven design, highlighting that system mechanism arises from coordinated interactions rather than isolated parameters. A central finding is that the primary limitation for ML in this field is not model capability, but domain shift and contextual heterogeneity across experimental conditions. While propulsion mechanisms can be classified with consistent agreement, indicating internal descriptor coherence, regression tasks such as velocity and lifetime prediction remain limited (R^2^ = 0.6 to 0.7), reflecting the multifactor and context-dependent nature of reported performance. These results demonstrate that current ML approaches are constrained by data structure, variability, and reporting practices rather than algorithmic complexity. Importantly, this work reframes ML as a structural and interpretive tool for mapping the design landscape of micro- and nanorobotics, enabling identification of descriptor coupling, reporting biases, and context-dependent trends. The data set itself is positioned as a reproducible resource to support future benchmarking and cross-study analysis. Looking forward, progress will depend on improved data practices, including standardized reporting of environmental conditions, material properties, and performance metrics, as well as the development of shared, high-quality data sets. Integrating data-driven approaches with physics-informed and bioengineering-aware models will be essential to achieve reliable, interpretable, and transferable predictions. Ultimately, establishing consistent data frameworks will enable ML to move from exploratory analysis toward predictive design in micro- and nanorobotics.

## Supplementary Material



## Data Availability

The data that support the findings of this study are available from the corresponding author upon reasonable request.

## Notes

Literature-derived data set of 100 micro- and nanorobot systems, including design, material, propulsion, experimental, and performance descriptors (Table S1). Machine-learning model configurations and tuned hyperparameters (Table S2). Model validation results, repeated train–test split performance, regression and classification metrics, and bootstrap confidence intervals (Table S3). (PDF)
