# Supplementary material for: Machine Learning-Enabled Meta-Analysis of Bioengineering Micro- and Nanorobots
Source: Nano Lett. 2026 Sep 27;26(39):13143–56. doi: 10.1021/acs.nanolett.6c02258 (PMC13651602; doi:10.1021/acs.nanolett.6c02258)
Supplement: Supplementary file 1 [file nl6c02258_si_001.pdf]

Supporting Information (SI)

Machine learning-enabled meta-analysis of bioengineering micro- and nanorobots

Ehsan Rahimi[1]*, Mario Palacios-Corella[2], Mohammad Rahimi[3]*, Lucky Odirile Mohutsiwa[4], Josep Puigmartí-Luis[2,5], Salvador Pané[4]*

[1]Faculty of Mechanical Engineering (ME), Delft University of Technology, Mekelweg 2, 2628 CD Delft, The Netherlands

[2]Departament de Ciència dels Materials i Química Física, Institut de Química Teòrica i Computacional, Universitat de Barcelona, Barcelona, 08028, Spain

[3]Department of Mechanical Engineering, McMaster University, Hamilton, ON L8S 4L7, Canada

[4]Multi-Scale Robotics Lab, Institute of Robotics and Intelligent Systems, Department of Mechanical and Process Engineering, ETH Zurich, Tannenstrasse 3, Zurich 8092, Switzerland

[5]Institució Catalana de Recerca i Estudis Avançats (ICREA), Pg. Lluís Companys 23, Barcelona 08010, Spain
Corresponding authors:

E.Rahimi:rahimi.ehn@gmail.com & M.Rahimi: rahimm19@mcmaster.ca, S. Pané: vidalp@ethz.ch

S1. Methodology and data acquisition

S1.1. Literature-derived dataset

An ML-oriented meta-analysis was performed based on published studies on bioengineering-relevant micro- and nanorobots, such as micro- and nanomotors or machines, which are summarized in **Table S1** along with descriptions of their corresponding experimental systems. A literature-derived database was constructed by systematically searching studies published between 2010 and 2026. The search strategy was designed to cover the broad design space of bioengineering-oriented micro- and nanorobotic systems, including microrobots, nanorobots, micromotors, nanomotors, microswimmers, nanoswimmers, biohybrid microrobots, and active matter-based microswimmers. These terms were combined with application-related keywords such as bioengineering, nanomedicine, targeted drug delivery, minimally invasive therapy, biosensing, diagnostics, biocompatibility, biodegradability, degradation, *in vitro*, *in vivo*, and biological media. To make the collected studies suitable for ML analysis, the search also included terms describing the design and physical properties, propulsion and actuation methods, biofunctionalization, and the broader design features of micro- and nanorobotic systems.

S1.2. Database preparation

The keywords were used individually and in different combinations to capture studies reporting material composition, shape, characteristic size, propulsion mechanisms, operating media, biological testing conditions, degradation behavior, measured signals, and performance outcomes. From the retrieved literature, relevant information was manually extracted and organized into a structured dataset. Each study was screened to identify commonly reported descriptors, including shape and characteristic size, material and chemical composition, propulsion mechanism, application or function, biological testing conditions, pH, temperature, degradability or lifetime assessment, measured signals, and performance outcomes such as instantaneous velocity or degradation behavior. Eligibility was assessed at the system level, and studies were included if they were peer-reviewed primary research articles describing a distinct micro- or nanorobot, motor, or swimmer relevant to bioengineering. The studies were also required to provide sufficient information on the propulsion mechanism, together with at least one design or material descriptor and one experimental-context or performance descriptor. Studies were excluded if they were reviews, editorials, or duplicate reports of the same experimental system. Purely theoretical or control-focused studies without extractable system-level observations, as well as records lacking the minimum required descriptor set, were also excluded. The search was designed to maximize coverage across actuation modalities and biomedical contexts rather than to constitute an exhaustive systematic review. Accordingly, the dataset is representative of the descriptor patterns captured by the stated search and eligibility criteria, but it should not be interpreted as a complete census of all published micro- and nanorobot studies.

S1.3. Descriptor selection

Qualitative descriptors were converted into numerical or categorical formats, while continuous variables were retained in their reported numerical form. The resulting dataset includes 100 distinct micro- and nanorobot systems drawn from 100 peer-reviewed studies and covers chemically propelled, magnetically actuated, acoustic driven, light-driven, and biohybrid platforms. The extracted descriptors include robot shape and size, material composition, propulsion mechanism, application, operating environment, degradability and lifetime, measured signals, and instantaneous velocity. Detailed representations of these quantitative and qualitative variables are provided in **Table S1**. This structured dataset converts heterogeneous narrative reports into semi-quantitative and categorical features suitable for ML-based analysis [1, 2], allowing relationships among design choices, experimental conditions, biological constraints, and

propulsion strategies to be investigated. This approach also reflects a practical limitation of the field: raw trajectory and imaging data are rarely shared, whereas summary descriptors and performance metrics are more consistently reported.

S2. Data representation and encoding of qualitative variables

The literature-derived dataset in **Table S1** contains mixed-type descriptors, including categorical variables such as shape, material composition, propulsion mechanism, biological medium, and measured observables, together with numerical variables such as pH, temperature, instantaneous velocity, and degradability/lifetime. To enable unified machine-learning analysis, all records were transformed into a structured feature matrix containing encoded qualitative variables and normalized quantitative variables. The i-th micro/nanorobot system can be represented as:

$$x^{(i)} = [x_1^{(i)}, x_2^{(i)}, ..., x_p^{(i)}] \in \mathbb{R}^p \qquad (Eq.\,S1)$$

where $p$is the total number of input descriptors after preprocessing. For nominal categorical descriptors without intrinsic ordering, such as propulsion mechanism, biological medium, measured observables, and application type, one-hot encoding was used. In this representation, each category is converted into a separate binary indicator variable:

$$z_{ijk} = \begin{cases} 1, & \text{if sample } i \text{ belongs to category } k \text{ of descriptor } j \\ 0, & \text{otherwise} \end{cases} \qquad (Eq.\,S2)$$

where z_{ijk} denotes the binary encoded value for category k of descriptor j. This avoids imposing artificial ordinal relationships among nominal classes such as magnetic, catalytic, acoustic, and light-driven propulsion. Grouped numerical labels were used only for descriptors with meaningful, ordered or semi-quantitative interpretation. Nominal descriptors, including propulsion mechanism, biological medium, measured observables, and application type, were encoded using one-hot encoding to avoid artificial ordinal relationships.

S2.1. Quantitative variable normalization

For each quantitative variable N_j, such as pH, temperature, velocity, or lifetime, normalization was applied to place variables on comparable scales:

$$\tilde{x}_{ij} = \frac{x_{ij} - \mu_j}{\sigma_j} \qquad (Eq. S3)$$

where μ_j and σ_j are the mean and standard deviation of the variable j, respectively. If a min-max scaling scheme was preferred for a given task, the transformed value was expressed as:

$$\tilde{x}_{ij} = \frac{x_{ij} - x_j^{min}}{x_j^{max} - x_j^{min}} \qquad (Eq. S4)$$

Depending on the requirements of the downstream model. The detailed configuration and tuned hyperparameters of all machine learning models are provided in **Table S2**.

S.3. Data handling and model validation

Several studies reported velocity or lifetime as a range rather than a single value. To ensure consistency, the arithmetic midpoint of each range was used as the representative value, while individually reported values were retained unchanged. Systems lacking the target variable were excluded from the corresponding regression task rather than assigned artificial values. Model uncertainty arising from the relatively small dataset was evaluated using repeated stratified 80/20 train–test splits across multiple random seeds, with preprocessing and hyperparameter tuning conducted only within each training partition. Nonparametric bootstrap refitting was also performed to estimate 95% confidence intervals. As summarized in **Table S3**, the propulsion classification model achieved a held-out (R^2) of 0.7619, with RMSE and MAE values of 1.3371 and 0.672, respectively. Across repeated random splits, its mean performance was (0.6868 ± 0.0845), and the bootstrap 95% confidence interval ranged from 0.6211 to 0.7195. The propulsion classes were imbalanced, with magnetic and catalytic/chemical systems accounting for 45 and 40 of the 100 entries, respectively, compared with eight light-driven, five ultrasound-driven, and two unassigned systems. The velocity regression model achieved a held-out of 0.6374, an RMSE of 56.4833, and an MAE of 24.8879, with a repeated-split result of 0.5811 ± 0.0720. Because oversampling very small minority classes may introduce unstable or artificial observations, classification performance was assessed using imbalance-aware metrics and class-weighted training. Balanced accuracy and macro- or weighted-F1 should therefore be reported alongside the overall classification results. Similarly, the lifetime regression model showed a held-out of 0.6064, an RMSE of 95.860, and an MAE of 42.912, and a bootstrap confidence interval of 0.5172 to 0.6338. Overall, these results indicate moderate variability across data partitions but generally consistent model behaviour.

Table S1. Summary of ~100 reported micro- and nanorobot systems compiled from peer-reviewed studies, including design descriptors, propulsion mechanisms, experimental environment (biological medium, pH, temperature, in vitro/in vivo), and performance metrics (measured observables, velocity, and where available, lifetime or degradability). The table provides the structured input for the illustrative machine-learning analyses presented in this work.

Shape & characteristic size	Material/chemical composition	Propulsion/motion mechanism	Application/ Functionality	Actuation classification				Degradability & lifetime assay	Measured signals/observables	Instantaneous velocity (μm s⁻¹)	*Ref.*
				Biological medium		Environmental conditions					
				In/ex vivo	*In vitro*	pH	Temperature				
Microtubular	PANI/Zn	Catalytic/chemical	Biomedical	-	HCl	0.2 - 1	23 °C	2 min	Video-tracked trajectory	20 to 1050	3
Microtubular	PEDOT/MnO_2/Ag	Catalytic/chemical	Anti-bacterial	-	0.2-5% H_2O_2		23 °C	10 min	Video-tracked trajectory	122 to 562	4
Janus microball	Al-Ga/Ti	Catalytic/chemical	Biomedical	Cell culture media	Water, PBS, and human serum	7	23 °C	5 min	Video-tracked trajectory	100 to 3000	5
Janus microball	Mg/Pt	Catalytic/chemical	Biomedical	-	PBS+0.5 mNaHCO$_3$ and 5 wt% PVP	7	37 °C	70 s	Video-tracked trajectory	59 to 92	6
Microhelix/roller	Fe	Magnetic	Captured cancer cell	RPMI 1640 Medium	-	7.4	23 °C	20 min	Magnetic field rotating/response	42	7
Janus microball	Zn/Pt	Catalytic/chemical	Anti-bacterial (E.*coli* cell)	Culture medium	-	7	23 °C	-	Video-tracked trajectory	0.1 to 0.15	8
Microtubular	Bi/Ni/Pt	Magnetic	Drug delivery	-	0.5% SDS+1% H_2O_2	-	37 °C	600 min	Magnetic field rotating/response and Video-tracked trajectory	102 to 384	9
Microparticles	Ag and MnO_2	Catalytic/chemical	Biomedical	-	0.1-12% H_2O_2	-	23 °C	-	Video-tracked trajectory	50 to 100	10
Janus microball	SiO_2/Pt	Catalytic/chemical	Biomedical	-	1-10% H_2O_2	-	23 °C	-	Video-tracked trajectory	4 to 16	11
Micro tubular and helical	CoNiReP	Magnetic	Drug delivery	-	Water	-	23 °C	-	Magnetic field rotating/response and Video-tracked trajectory	0.4 to 3.7	12
Micro and nanotubular	Pt	Catalytic/chemical	Biomedical	-	1-10% H_2O_2	-	23 °C	-	Video-tracked trajectory	200 to 1000	13
Nanotube	Ni/Chi/Au	Magnetic	Drug delivery	-	PBS	6 -7.4	23 °C	-	Video-tracked trajectory	-	14
Nanowire	Fe/C	Magnetic	Drug delivery	-	Water	-	23 °C	-	Magnetic field rotating/response and Video-tracked trajectory	100 to 80	15
Nanowire	Co/Au	Magnetic	Biomedical	-	-	-	-	-	Magnetic field rotating/response	-	16
Nanowire	Ni/Ppy/P	Magnetic	Drug delivery	-	Tris HCl	8.5	23 °C	-	Magnetic field rotating/response and Video-tracked trajectory	5.5, 0.91 (translational)	17
Nanoparticles	Fe	Magnetic	Cancer theranostics	Culture medium	Buffer media	7.4, 6.5, 5.4	37 °C	2880 min	Magnetic field rotating/response, MRI and magnetic targeting	-	18
Nanoparticles	Fe/PEG	Magnetic	Cancer theranostics	Culture medium	PBS	7.4	37 °C	-	Magnetic field rotating/response	-	19
Nanoparticles	Fe/Gd_2O_3	Magnetic	Biomedical (cell viability)	Culture medium	PBS	7.4	37 °C	-	Magnetic field rotating/response, In Vivo MRI	-	20
Nanoparticles	Fe/Chi, PEG or PEI	Magnetic	Cancer therapy	Culture medium	Buffer media	7.4	37 °C	-	Magnetic field rotating/response, MRI	-	21
Nanoparticles	Fe/Au	Magnetic	Hepatoma imaging	reticuloendot helial organs (liver)	-	-	-	-	Magnetic field rotating/response, MRI and computed tomography (CT)	-	22
Nanoparticles	CFO and NFO	Magnetic	Biomedical	Cell culture medium	-	7.4	37 °C	1440 min	Magnetic field rotating/response	-	23

Nanoparticles	Ag/Au	Light	Photothermal Therapy	*in vitro* in tumor cells	*in vivo* in tumors	7.4	37 °C	36000 min	Infrared thermal imaging	-	24
Nanoparticle	Citrate-coated iron oxide	Magnetic	Therapeutic	-	Acidic citrate buffer	4.7	37 °C	43200 min	MRI imaging of the inflammation process	-	25
Nanoparticle	CFO	Magnetic	Cancer therapeutic	Livers and spleens	Acidic citrate buffer	4.7	37 °C	50400 min	MRI imaging of the inflammation process	-	26
Nanoparticle	Iron oxide	Magnetic	Diagnostic and therapeutic	Livers and spleens	-	7.2	37 °C	43200 min	MRI imaging of the inflammation process	-	27
Nanoparticle	Iron oxide	Magnetic	Diagnostic and therapeutic	Astrocytes brain cell	-	7.2	37 °C	-	MRI imaging	-	28
Nanoparticle	Iron oxide	Magnetic	Therapeutic	Human Mesenchym al stem cells (Lonza)	Acidic citrate buffer	4	37 °C	31680 min	MRI imaging	-	29
Nanoparticle	Fe/Au	Magnetic	Therapeutic	liver, spleen, kidney, and lung	Acidic citrate buffer	4.7-7.4	37 °C	43200 min	MRI imaging and Electron spin resonance (ESR)	-	30
Nanoparticle	Cu and Ag	-	Diagnostic and therapeutic	-	100 mM $NaClO_4$+ 1 mM HCl	3	23 °C	360-420 s	Surface plasmon microscopy (SPM)	-	31
Nanowire	Iron oxide	Magnetic	Cancer therapeutic	NIH/3T3 fibroblast cells	-	1.4-9.1	37 °C	1440 min	Optical and electron microscopy,	-	32
Janus microball	Pt/TiO_2	Catalytic/chemical	Blood vessel	-	5 wt% H_2O_2	7	23 °C	-	Video-tracked trajectory	20 to 140	33
Microtubes	Cu/Pt and Pt/Fe/Cr/Pt	Catalytic/chemical	Blood vessel	-	Distilled water (DS), DS+1.15 %SDS and DS+ 1.15%SD S+ 7.76% H_2O_2	7	23 °C	10080 min	Video-tracked trajectory	200	34
Nanorods/helice	Cu/Ti/HfO_2 and Co/Ti/HfO_2	Magnetic	Biomedical	-	PBS	3.7	23 °C	840-960 min	ferromagnetic, plasmonic, and chiral properties	-	35
Microtubes	Pt	Catalytic/chemical	Blood vessel	-	5 wt% H_2O_2	7	25 °C	36000 min	Video-tracked trajectory	50 to 140	36
Micropillars	Thick Au	-	Diagnostic and therapeutic	-	PBS+ 1g/L BSA	7.4	37 °C	-	-	-	37
Micro-spherical (60 µm)	Fe–Co NPs	Magnetic	magnetic steering for tumor chemoemboliza tion	Right or left liver lobes	0.9% NaCl	-	-	-	MRI imaging of therapeutic magnetic microcarriers	-	38
Micro-spherical (50 µm)	Fe–Co NPs	Magnetic	Targeted drug delivery/chemo embolization	Right or left liver lobes	0.9% NaCl	-	-	-	MRI imaging of therapeutic magnetic microcarriers	-	39
2D shapes (240–600 µm)	Fe MPs	Magnetic	Drug release	-	PBS	7.4	37 °C	60440 min	Magnetic field response and Video-tracked trajectory	1500 to 3000	40
Micro-helical (5–40 × 30–120 µm)	Iron oxide NPs	Magnetic	Drug release	-	PBS	7.4	37 °C	240 min	Video-tracked trajectory	40 to 50	41
Micro-helical	Fe NPs	Magnetic	AMF-triggered drug release and hyperthermia	Cell culture medium (HCT116 cells)	PBS	7.4	37 °C	1800 min	Magnetic field response and Video-tracked trajectory	80 to 110	42
Micro-helical (5–20 × 20–50 µm)	Iron oxide NPs	Magnetic	Enzyme-triggered drug release	Cell culture medium +	-	7.4	37 °C	7200 min	Magnetic field response and Video-tracked trajectory	3.3	43

				MMP-2 enzyme							
Micro-helical	Iron oxide	Magnetic	cell-secreted proteases	Cell culture medium (HaCaT cells)	PBS+ glucose	7.4	37 °C	10080 min	Magnetic field response and Video-tracked trajectory	15	44
Micro-helical	CFO-BFO	Magnetic	Cell delivery and electrostimulation	Cell culture medium (SH-SY5Y cells)	PBS	7.4	37 °C	10080 min	Magnetic field response and Video-tracked trajectory	40 to 250	45
Micro-Star-like (20 µm)	Iron oxide	Magnetic	pH-triggered drug release	-	PBS + human saliva	7.4	37 °C	1440 min	Magnetic field response and Video-tracked trajectory	250	46
Micro-spherical (50–100 µm)	Iron oxide	Magnetic	Ultrasound-triggered drug release	-	Sodium alginate	-	-	-	Magnetic field response and Video-tracked trajectory	20 to 60	47
Micro-pherical (800 µm)	Au-Ni-Au	Magnetic	Drug release	-	PBS	7.4	-	-	Magnetic field response and Video-tracked trajectory	1000	48
Micro-helical (5 × 20 µm)	Iron oxide NPs	Magnetic	Light-triggered drug release (photocleavable linker)	Cell culture medium (SKBR3 breast cancer cell)	-	7.4	37 °C	12960 min	Magnetic field response and Video-tracked trajectory	3.3	49
Nano-wires	Au-Ni-Au	Magnetic	Drug release	-	PBS	-	23 °C	-	Magnetic field response and Video-tracked trajectory	14	50
Micro-pillar	Iron oxide + DNA nanotubes	Magnetic	–	Biomedical	TBE buffer	-	-	-	Magnetic field response and Video-tracked trajectory	0.1 to 0.5	51
Micro-helical (400 nm × 5 µm)	Ni/Au-platelet membranes	Magnetic	Biodetoxification	Cell culture medium	-	-	23 °C	-	Magnetic field response and Video-tracked trajectory	6	52
Micro-helical (10–50 × 100–200 µm)	Iron oxide	Magnetic	In vivo fluorescence and MRI	athymic mice tissues	DPBS	7.4	37 °C	1440 min	Fluorescence-based imaging, MRI, magnetic field response, and Video-tracked trajectory	10 to 100	53
Micro-helical	Iron oxide	Magnetic	Drug transport and release	-	DPBS	-	37 °C	4320 min	Magnetic field response and Video-tracked trajectory	-	54
Micro-helical	Pd-Au NPs	Magnetic	Light-triggered chemo-phototherapy	Cell culture medium (Cancer cells)	-	-	37 °C	20 min	Magnetic field response and Video-tracked trajectory	50 to 500	55
Micro-helical	Barium titanate	Magnetic	Piezoelectric cell differentiation	Cell culture medium (PC12 cells)	DPBS	7.4	37 °C	7200 min	Magnetic field response and Video-tracked trajectory	333	56
Micro-oval	Iron oxide	Magnetic	Toxin detection (carbon dots)	bacteria culture medium	PBS and DMEM	-	-	-	Magnetic field response and Video-tracked trajectory	8.3	57
Micro-tubular (3 × 20 µm)	Pt–ZnO	Light	Cargo transport	-	10% H_2O_2	-	-	-	UV light response, Video-tracked trajectory	250 to 470	58
Micro-rod-like (1.5 × 3.4 µm)	ZnO-Au	Light	Cargo transport	-	1% H_2O_2	-	-	-	UV irradiation, Video-tracked trajectory	10	59
Micro-spherical (50–120 nm)	Silica-Au	Light	Drug delivery	-	PBS+FBS	7.4	37 °C	-	NIR laser irradiation response, Video-tracked trajectory	8	60
Micro-spherical (5 µm)	Chitosan-heparin-Au	Ultrasound	Photothermal therapy	Cell culture medium, blood	PBS, serum	7.4	37 °C	1440 min	NIR laser irradiation response, Video-tracked trajectory	10 to 200	61
Micro-rod (400 nm × 3 µm)	Au-Red blood cell (RBC) membrane	Ultrasound	Toxin adsorption/neutralization	Includes undiluted whole blood	PBS	-	-	-	Ultrasound field, Video-tracked trajectory	32	62
Micro-rod/pillar	Au-Hybrid RBC + platelet (PL) membrane	Ultrasound	Simultaneous bacteria binding + toxin neutralization	Whole blood/ contaminate	PBS	-	-	-	Ultrasound field, Video-tracked trajectory	38	63

				d biological samples							
RBC (6–8 µm)	RBCs-Iron oxide	Ultrasound	Magnetic steering, fluorescence, and drug release	-	PBS, serum	7.4	37 °C	-	Ultrasound field, Video-tracked trajectory	10 to 60	64
Micro-Janus-spherical (20–50 µm)	RBC-Mg or Zn	Catalytic/chemical	Detoxification (RBC membrane)	-	0.08 M NaCl+albumin, HEPES buffer, PBS	7.4, 8.5	23 °C	-	Video-tracked trajectory	165	65
Micro-Janus	Mg/Pt	Catalytic/Chemical	Temperature-triggered drug release (PNIPAM)	-	Tris-HCl buffered solution	7.4	20-37 °C	60 s	Video-tracked trajectory	95 to 105	66
Micro-Janus	Mg/Au/polymer	Catalytic/Chemical	In vivo stomach-targeted drug delivery	mouse stomach	PBS	1.3-7	-	-	Fluorescence imaging,, Video-tracked trajectory	60	67
Micro-Janus	Mg/TiO_2/polymer	Catalytic/Chemical	In vivo stomach-targeted drug delivery and In vivo antibacterial therapy	Cell culture medium + 5% FBS	PBS	0.75-1.75	37 °C	-	Fluorescence imaging, Video-tracked trajectory	120	68
Micro-tubes-(2–10 × 10–20 µm)	Zn	Catalytic/Chemical	In vivo stomach-targeted drug delivery	ICR male mice	Gastric fluid (2.0 g NaCl and 7 mL of HCl (12 M) + 1L water)	1.2	37 °C	720 min	Video-tracked trajectory	60	69
Micro-tubes	Au/Mg	Catalytic/Chemical	In vivo stomach-targeted drug delivery	Old ICR male mice	Gastric fluid	1-3	37 °C	150 min	Video-tracked trajectory	60	70
Micro-tubes	Zn/Fe	Catalytic/Chemical	Cargo transport and release	old ICR male mice	Gastric fluid	1.2	37 °C	1 min	Fluorescence imaging, Video-tracked trajectory	34	71
Micro-conical (5 × 15 µm)	Zn/Si	Catalytic/Chemical	Cargo transport and release	-	0.7 M HCl	-	24 °C	20 s	Video-tracked trajectory	110 to 180	72
Nano-Janus (65 nm)	Pt/Cr	Catalytic/Chemical	Drug release	-	1% H_2O_2	-	24-37 °C	-	Video-tracked trajectory	1.8 to 2.4	73
Micro-Janus (2 µm)	Ni/SiO_2	Catalytic/Chemical	Drug release	-	1.5 wt % H_2O_2	-	22 °C	-	Video-tracked trajectory	-	74
Vesicular (350 nm)	semicrystalline poly(ε-caprolactone)-PCL-Pt	Catalytic/Chemical	Drug release	-	50% H_2O_2	1	37 °C	720 min	Confocal imaging, Video-tracked trajectory	40 to 50	75
Vesicular	Copolymer-Pt	Catalytic/Chemical	Redox-triggered drug release	-	H_2O_2	-	37 °C	-	Video-tracked trajectory	5 to 45	76
Nano-tublar (150 × 900 nm)	PEG-PDLLA polymersomes	Catalytic/Chemical	Drug release	-	H_2O_2	-	24 °C	-	Fluorescence imaging, Video-tracked trajectory	-	77
Nano-tubular (600 nm × 10 µm)	Chitosan-alginate-Pt NPs	Catalytic/Chemical	Magnetic steering, ultrasound-triggered drug release	-	1-15 % H_2O_2	-	37 °C	-	Video-tracked trajectory	5 to 70	78

Micro-tubular (5 × 10 µm)	bovine serum albumin/poly-l-lysine (PLL/BSA)	Catalytic/Chemical	Light-triggered drug release	Hela cells	0.5% H_2O_2	7.4	37 °C	720 min	NIR irradiation, Video-tracked trajectory	4 to 59	79
Micro-tubular (1 × 24 µm)	avidin–biotin	Catalytic/Chemical	Drug release	-	5 wt % H_2O_2	3–7.5	37 °C	-	Video-tracked trajectory	30 to 100	80
Micro-tubular	avidin/biotinylated urease	Catalytic/Chemical	Drug release	-	aqueous solution of protease mixture	3	37 °C	120 min	Video-tracked trajectory	2.7 to 10	81
Micro-tubular	avidin/Pt	Catalytic/Chemical	Specific molecular trapping	-	aqueous solution of protease mixture	7.5	37 °C	120 min	Fluorescence imaging, video-tracked trajectory	138	82
Nano-Janus	Pt/$CaCO_3$@HA-CB	Catalytic/Chemical	drug delivery	-	PBS	6.5	37 °C	120 min	Confocal imaging, video-tracked trajectory	4 to 14	83
Micro-spherical (2–20 µm)	Bacterial/polystyrene	Catalytic/Chemical	Chemotaxis, tumor targeting	-	water	7	25°C	-	Video-tracked trajectory	1.2 to 3.3	84
Micro-spherical (2µm)	Bacteria/polystyrene	Catalytic/Chemical	Chemotaxis, tumor targeting	Cell culture medium (CT-26 cells)	-	-	37 °C	-	Video-tracked trajectory	0.16 to 0.5	85
Micro-spherical (2µm)	Bacteria/Pt or Au or Ti or Fe	Catalytic/Chemical	bacteria attachment	PBS+ E. coli	-	7.4	37 °C	-	Video-tracked trajectory	0.4 to 0.7	86
Nanoparticles (40–600 nm)	Bacteria/polystyrene	Catalytic/Chemical	In vivo drug delivery	Cell culture medium (Tumour cells)	-	-	37 °C	30 min	Video-tracked trajectory	-	87
Nanorods (10–20 × 40–60 nm)	Bacteria/Au	Light	NIR imaging, photothermal therapy	tumor tissues (lung, liver, heart, spleen, and kidney)	-	-	37 °C	-	NIR light excitation, Video-tracked trajectory	-	88
Micro-spherical (10µm)	Bacteria/poly(ethylene glycol)	Catalytic/Chemical	Diagnosis and therapy	-	Water	-	37 °C	-	Video-tracked trajectory	0.1 to 0.4	89
Micro-spherical (10–20 µm)	Bacteria/Alginate	Magnetic	Magnetic steering, chemotaxis	Flagellate bacteria media	-	-		-	Magnetic field response, Video-tracked trajectory	1 to 12	90
Micro-spherical (5–15 µm)	Bacteria/Liposomes/ microemulsions	Catalytic/Chemical	Drug delivery	Cell culture medium	PBS	-	37 °C	-	Video-tracked trajectory	4.5 to 71	91
Nano-spherical (200 nm)	Magnetotactic bacteria/Nanoliposomes	Magnetic	Magnetic steering, drug delivery	Cell culture medium (MC-1)	PBS	7.4	37 °C	-	Video-tracked trajectory	80 to 200	92
RBC (5 µm)	RBCs + bacteria/Iron oxide	Magnetic	pH-triggered drug delivery, NIR inactivation	Cell culture medium (*E. coli* MG1655)	-	3.1-9.2	37 °C	-	NIR light–triggered hyperthermia, Video-tracked trajectory	10.2	93
Micro-spherical (5 µm)	Algae/polystyrene	Catalytic/Chemical	Phototaxis	Cell culture medium	PBS	-	-	-	Video-tracked trajectory	100 to 200	94
Micro-spherical (5–10 nm)	Sperm cells/Cd-Se NPs / iron oxide / Pt	Catalytic/Chemical	Chemotaxis, chemotherapy (DOX)	Culture medium (SKOV-3 cells)	-	4	37 °C	-	Video-tracked trajectory	217	95
Micro-spheres	TiO_2/PLGA/Fe–Se@chitosan-coated Mg	Catalytic/Chemical	Mineral delivery (iron deficiency anemia)	-	gastric fluid	6.5	-	-	Video-tracked trajectory	120 to 164	96
Nano-spheres	Mo–Pt–Pd	Catalytic/Chemical	ROS scavenging	-	water, (PBS), Dulbecco'	5-9	37 °C	-	Video-tracked trajectory	1500	97

			(traumatic brain injury)		s modified Eagle medium (DMEM), and artificial cerebrospinal fluid (ACSF)						
Micro-spheres	PDA/Fe_3O_4-coated microalgae	Magnetic	Photothermal therapy (bacterial infection)	MDR *KP* colonies	-	-	37 °C	30 min	Magnetic field response, video-tracked trajectory	4 to 123	98
Microrods	CaO_2	Ultrasound	Sonodynamic therapy (thrombus)	-	blood clots in simulated blood vessels	7.4	37 °C	120 min	Ultrasonication field response, video-tracked trajectory	2.3	99
Nano-spheres	Pt/Si	Light	Photothermal therapy (tumor) and drug delivery	Cell culture medium (MCF-7 cells)	-	7.4	37 °C	-	NIR irradiation response, Video-tracked trajectory	0.5 to 12.7	100
Microsphere	Au/α-Si@SiO_2	Light	Anti-bacterial		70 µm MFc and 1 m NaCl	7.4	37 °C	-	Video-tracked trajectory	50	101
Microsphere	PS@PDA-ICG	Light	Photothermal therapy (tumor)	melanoma tumor mice.	-	-	37 °C	-	NIR irradiation response, Video-tracked trajectory	-	102

Table S2. The tuned hyperparameter of ML approaches in this study.

Target	Task type	Model	Hyperparameters
Instantaneous velocity (μm. s^{-1})	Regression	GBR	{"model_subsample": 0.7, "model_n_estimators": 100, "model_max_depth": 4, "model_learning_rate": 0.01}
		XGB	{"model_subsample": 0.7, "model_n_estimators": 200, "model_max_depth": 5, "model_learning_rate": 0.01, "model_colsample_bytree": 0.7}
		KNN	{"model_weights": "uniform", "model_p": 2, "model_n_neighbors": 15}
Degradability & lifetime assay	Regression	Ridge	{"model_alpha": 621.0169}
		ETs	{"model_n_estimators": 700, "model_min_samples_split": 2, "model_min_samples_leaf": 4, "model_max_features": "log2", "model_max_depth": 40}
		ElasticNet	{"model_l1_ratio": 0.05, "model_alpha": 6.72335}
Propulsion/motion mechanism	Classification	RF	{"model_n_estimators": 700, "model_min_samples_split": 2, "model_min_samples_leaf": 4, "model_max_features": "log2", "model_max_depth": 40}
		SVR	{"model_kernel": "rbf", "model_gamma": "scale", "model_epsilon": 0.3727, "model_C": 0.16237}

Table S3. Robustness and uncertainty analysis of the propulsion classification, velocity regression, and lifetime regression models, including held-out performance, repeated random-split mean ± standard deviation (SD), bootstrap 95% confidence intervals (CI), balanced accuracy, and weighted F1-score.

**Task**	**Current held-out result of R^2**	**Repeated split**	**Repeated-split mean ± SD**	**Bootstrap 95% CI**	**Balanced accuracy**	**F1_score**
Propulsion classification	Accuracy = 76.19%	20	76.2% ± 0.085	[62.11%, 81.95%]	66.14%	72.36%
Velocity regression	R^2 = 0.6374<br>RMSE= 56.4833<br>MAE= 24.8879	20	0.64 ± 0.072<br>56.48 ± 5.380<br>24.89 ± 2.663	[0.5654, 0.6714]<br>[42.5269, 86.0533]<br>[15.8416, 43.9325]	-	-
Lifetime regression	R^2 = 0.6064<br>RMSE= 95.860<br>MAE= 42.912	20	0.61 ± 0.054<br>95.86 ± 9.22<br>42.91 ± 5.83	[0.5172, 0.6338]<br>[81.7622, 113.715]<br>[34.5172, 59.9631]	-	-

References

(1) Lodhi, A.; Jindal, A.; Tomar, D.; Kumar, A.; Sharma, K. Artificial Intelligence in Nanocarrier Design and Drug Delivery via Nanorobotics-Based Personalised Medicine for Cancer Diagnostics and Therapy. In *Generative Intelligence in Healthcare*, CRC Press, 2025; pp 91-124.

(2) Deshmukh, S. S.; Yadav, K. S. Next-gen cancer therapy: The convergence of artificial intelligence, nanotechnology, and digital twin. *Next Nanotechnology* **2025**, *8*, 100286.

(3) Gao, W.; Uygun, A.; Wang, J. Hydrogen-bubble-propelled zinc-based microrockets in strongly acidic media. *Journal of the American Chemical Society* **2012**, *134* (2), 897-900.

(4) Liu, W.; Ge, H.; Ding, X.; Lu, X.; Zhang, Y.; Gu, Z. Cubic nano-silver-decorated manganese dioxide micromotors: enhanced propulsion and antibacterial performance. *Nanoscale* **2020**, *12* (38), 19655-19664.

(5) Gao, W.; Pei, A.; Wang, J. Water-driven micromotors. *ACS nano* **2012**, *6* (9), 8432-8438.

(6) Mou, F.; Chen, C.; Ma, H.; Yin, Y.; Wu, Q.; Guan, J. Self-Propelled Micromotors Driven by the Magnesium–Water Reaction and Their Hemolytic Properties. *Angewandte Chemie International Edition* **2013**, *52* (28), 7208-7212.

(7) Alcântara, C. C.; Kim, S.; Lee, S.; Jang, B.; Thakolkaran, P.; Kim, J. Y.; Choi, H.; Nelson, B. J.; Pané, S. 3D Fabrication of fully iron magnetic microrobots. *Small* **2019**, *15* (16), 1805006.

(8) Yoshizumi, Y.; Okubo, K.; Yokokawa, M.; Suzuki, H. Programmed transport and release of cells by self-propelled micromotors. *Langmuir* **2016**, *32* (37), 9381-9388.

(9) Beladi-Mousavi, S. M.; Khezri, B.; Krejčová, L.; Heger, Z. k.; Sofer, Z. k.; Fisher, A. C.; Pumera, M. Recoverable bismuth-based microrobots: capture, transport, and on-demand release of heavy metals and an anticancer drug in confined spaces. *ACS applied materials & interfaces* **2019**, *11* (14), 13359-13369.

(10) Wang, H.; Zhao, G.; Pumera, M. Beyond platinum: Bubble-propelled micromotors based on Ag and MnO2 catalysts. *Journal of the American Chemical Society* **2014**, *136* (7), 2719-2722.

(11) Ma, X.; Jang, S.; Popescu, M. N.; Uspal, W. E.; Miguel-López, A.; Hahn, K.; Kim, D.-P.; Sánchez, S. Reversed janus micro/nanomotors with internal chemical engine. *ACS nano* **2016**, *10* (9), 8751-8759.

(12) Schuerle, S.; Pané, S.; Pellicer, E.; Sort, J.; Baró, M. D.; Nelson, B. J. Helical and tubular lipid microstructures that are electroless-coated with CoNiReP for wireless magnetic manipulation. *Small* **2012**, *8* (10), 1498-1502.

(13) Wang, H.; Moo, J. G. S.; Pumera, M. From nanomotors to micromotors: The influence of the size of an autonomous bubble-propelled device upon its motion. *ACS nano* **2016**, *10* (5), 5041-5050.

(14) Hoop, M.; Mushtaq, F.; Hurter, C.; Chen, X.-Z.; Nelson, B. J.; Pané, S. A smart multifunctional drug delivery nanoplatform for targeting cancer cells. *Nanoscale* **2016**, *8* (25), 12723-12728.

(15) Zeeshan, M. A.; Pané, S.; Youn, S. K.; Pellicer, E.; Schuerle, S.; Sort, J.; Fusco, S.; Lindo, A. M.; Park, H. G.; Nelson, B. J. Graphite coating of iron nanowires for nanorobotic applications: Synthesis, characterization and magnetic wireless manipulation. *Advanced Functional Materials* **2013**, *23* (7), 823-831.

(16) Jang, B.; Pellicer, E.; Guerrero, M.; Chen, X.; Choi, H.; Nelson, B. J.; Sort, J.; Pané, S. Fabrication of segmented Au/Co/Au nanowires: insights in the quality of Co/Au Junctions. *ACS Applied Materials & Interfaces* **2014**, *6* (16), 14583-14589.

(17) Mushtaq, F.; Torlakcik, H.; Hoop, M.; Jang, B.; Carlson, F.; Grunow, T.; Läubli, N.; Ferreira, A.; Chen, X. Z.; Nelson, B. J. Motile piezoelectric nanoeels for targeted drug delivery. *Advanced Functional Materials* **2019**, *29* (12), 1808135.

(18) Zhang, C.; Bu, W.; Ni, D.; Zhang, S.; Li, Q.; Yao, Z.; Zhang, J.; Yao, H.; Wang, Z.; Shi, J. Synthesis of iron nanometallic glasses and their application in cancer therapy by a localized Fenton reaction. *Angewandte Chemie* **2016**, *128* (6), 2141-2146.

(19) Fang, C.; Bhattarai, N.; Sun, C.; Zhang, M. Functionalized nanoparticles with long-term stability in biological media. *Small* **2009**, *5* (14), 1637-1641.

(20) Zhou, Z.; Huang, D.; Bao, J.; Chen, Q.; Liu, G.; Chen, Z.; Chen, X.; Gao, J. A synergistically enhanced T1–T2 dual-modal contrast agent. *Advanced materials* **2012**, *24* (46), 6223-6228.

(21) Kievit, F. M.; Stephen, Z. R.; Wang, K.; Dayringer, C. J.; Sham, J. G.; Ellenbogen, R. G.; Silber, J. R.; Zhang, M. Nanoparticle mediated silencing of DNA repair sensitizes pediatric brain tumor cells to γ-irradiation. *Molecular oncology* **2015**, *9* (6), 1071-1080.

(22) Kim, D.; Yu, M. K.; Lee, T. S.; Park, J. J.; Jeong, Y. Y.; Jon, S. Amphiphilic polymer-coated hybrid nanoparticles as CT/MRI dual contrast agents. *Nanotechnology* **2011**, *22* (15), 155101.

(23) Lima, D. b. R.; Jiang, N.; Liu, X.; Wang, J.; Vulcani, V. A.; Martins, A.; Machado, D. S.; Landers, R.; Camargo, P. H.; Pancotti, A. Employing calcination as a facile strategy to reduce the cytotoxicity in CoFe2O4 and NiFe2O4 nanoparticles. *ACS applied materials & interfaces* **2017**, *9* (45), 39830-39838.

(24) Espinosa, A.; Curcio, A.; Cabana, S.; Radtke, G.; Bugnet, M.; Kolosnjaj-Tabi, J.; Péchoux, C.; Alvarez-Lorenzo, C.; Botton, G. A.; Silva, A. K. Intracellular biodegradation of Ag nanoparticles, storage in ferritin, and protection by a Au shell for enhanced photothermal therapy. *ACS nano* **2018**, *12* (7), 6523-6535.

(25) Volatron, J.; Carn, F.; Kolosnjaj-Tabi, J.; Javed, Y.; Vuong, Q. L.; Gossuin, Y.; Ménager, C.; Luciani, N.; Charron, G.; Hémadi, M. Ferritin protein regulates the degradation of iron oxide nanoparticles. *Small* **2017**, *13* (2), 1602030.

(26) Volatron, J.; Kolosnjaj-Tabi, J.; Javed, Y.; Vuong, Q. L.; Gossuin, Y.; Neveu, S.; Luciani, N.; Hémadi, M.; Carn, F.; Alloyeau, D. Physiological remediation of cobalt ferrite nanoparticles by ferritin. *Scientific reports* **2017**, *7* (1), 1-10.

(27) Levy, M.; Luciani, N.; Alloyeau, D.; Elgrabli, D.; Deveaux, V.; Pechoux, C.; Chat, S.; Wang, G.; Vats, N.; Gendron, F. Long term in vivo biotransformation of iron oxide nanoparticles. *Biomaterials* **2011**, *32* (16), 3988-3999.

(28) Geppert, M.; Hohnholt, M. C.; Nürnberger, S.; Dringen, R. Ferritin up-regulation and transient ROS production in cultured brain astrocytes after loading with iron oxide nanoparticles. *Acta Biomaterialia* **2012**, *8* (10), 3832-3839.

(29) Van de Walle, A.; Sangnier, A. P.; Abou-Hassan, A.; Curcio, A.; Hemadi, M.; Menguy, N.; Lalatonne, Y.; Luciani, N.; Wilhelm, C. Biosynthesis of magnetic nanoparticles from nano-degradation products revealed in human stem cells. *Proceedings of the National Academy of Sciences* **2019**, *116* (10), 4044-4053.

(30) Kolosnjaj-Tabi, J.; Javed, Y.; Lartigue, L.; Volatron, J.; Elgrabli, D.; Marangon, I.; Pugliese, G.; Caron, B.; Figuerola, A.; Luciani, N. The one year fate of iron oxide coated gold nanoparticles in mice. *ACS nano* **2015**, *9* (8), 7925-7939.

(31) Nizamov, S.; Kasian, O.; Mirsky, V. M. Individual detection and electrochemically assisted identification of adsorbed nanoparticles by using surface plasmon microscopy. *Angewandte Chemie International Edition* **2016**, *55* (25), 7247-7251.

(32) Safi, M.; Yan, M.; Guedeau-Boudeville, M.-A.; Conjeaud, H.; Garnier-Thibaud, V.; Boggetto, N.; Baeza-Squiban, A.; Niedergang, F.; Averbeck, D.; Berret, J.-F. Interactions between magnetic nanowires and living cells: uptake, toxicity, and degradation. *Acs Nano* **2011**, *5* (7), 5354-5364.

(33) Maric, T.; Nasir, M. Z. M.; Budanovic, M.; Alduhaish, O.; Webster, R. D.; Pumera, M. Corrosion of light powered Pt/TiO2 microrobots. *Applied Materials Today* **2020**, *20*, 100659.

(34) Zhao, G.; Khezri, B.; Sanchez, S.; Schmidt, O. G.; Webster, R. D.; Pumera, M. Corrosion of self-propelled catalytic microengines. *Chemical Communications* **2013**, *49* (80), 9125-9127.

(35) Jeong, H. H.; Alarcón-Correa, M.; Mark, A. G.; Son, K.; Lee, T. C.; Fischer, P. Corrosion-Protected Hybrid Nanoparticles. *Advanced Science* **2017**, *4* (12), 1700234.

(36) Maric, T.; Nasir, M. Z. M.; Wang, Y.; Khezri, B.; Pumera, M. Corrosion due to ageing influences the performance of tubular platinum microrobots. *Nanoscale* **2018**, *10* (3), 1322-1325.

(37) Rahimi, E.; Offoiach, R.; Deng, S.; Chen, X.; Pané, S.; Fedrizzi, L.; Lekka, M. Corrosion mechanisms of magnetic microrobotic platforms in protein media. *Applied Materials Today* **2021**, *24*, 101135.

(38) Pouponneau, P.; Leroux, J.-C.; Martel, S. Magnetic nanoparticles encapsulated into biodegradable microparticles steered with an upgraded magnetic resonance imaging system for tumor chemoembolization. *Biomaterials* **2009**, *30* (31), 6327-6332.

(39) Pouponneau, P.; Leroux, J.-C.; Soulez, G.; Gaboury, L.; Martel, S. Co-encapsulation of magnetic nanoparticles and doxorubicin into biodegradable microcarriers for deep tissue targeting by vascular MRI navigation. *Biomaterials* **2011**, *32* (13), 3481-3486.

(40) Kim, J.-y.; Jeon, S.; Lee, J.; Lee, S.; Lee, J.; Jeon, B. O.; Jang, J. E.; Choi, H. A simple and rapid fabrication method for biodegradable drug-encapsulating microrobots using laser micromachining, and characterization thereof. *Sensors and Actuators B: Chemical* **2018**, *266*, 276-287.

(41) Peters, C.; Hoop, M.; Pané, S.; Nelson, B. J.; Hierold, C. Degradable magnetic composites for minimally invasive interventions: device fabrication, targeted drug delivery, and cytotoxicity tests. *Advanced Materials* **2016**, *28* (3), 533-538.

(42) Park, J.; Jin, C.; Lee, S.; Kim, J.-Y.; Choi, H. Magnetically Actuated Degradable Microrobots for Actively Controlled Drug Release and Hyperthermia Therapy. *Advanced Healthcare Materials* **2019**, *8* (16), 1900213.

(43) Ceylan, H.; Yasa, I. C.; Yasa, O.; Tabak, A. F.; Giltinan, J.; Sitti, M. 3D-printed biodegradable microswimmer for theranostic cargo delivery and release. *ACS nano* **2019**, *13* (3), 3353-3362.

(44) Wang, X.; Qin, X. H.; Hu, C.; Terzopoulou, A.; Chen, X. Z.; Huang, T. Y.; Maniura-Weber, K.; Pané, S.; Nelson, B. J. 3D printed enzymatically biodegradable soft helical microswimmers. *Advanced Functional Materials* **2018**, *28* (45), 1804107.

(45) Dong, M.; Wang, X.; Chen, X. Z.; Mushtaq, F.; Deng, S.; Zhu, C.; Torlakcik, H.; Terzopoulou, A.; Qin, X. H.; Xiao, X. 3D-printed soft magnetoelectric microswimmers for delivery and differentiation of neuron-like cells. *Advanced Functional Materials* **2020**, *30* (17), 1910323.

(46) Hu, N.; Wang, L.; Zhai, W.; Sun, M.; Xie, H.; Wu, Z.; He, Q. Magnetically Actuated Rolling of Star-Shaped Hydrogel Microswimmer. *Macromolecular Chemistry and Physics* **2018**, *219* (5), 1700540.

(47) Rutkowski, S.; Si, T.; Gai, M.; Sun, M.; Frueh, J.; He, Q. Magnetically-guided hydrogel capsule motors produced via ultrasound assisted hydrodynamic electrospray ionization jetting. *Journal of colloid and interface science* **2019**, *541*, 407-417.

(48) Mair, L. O.; Chowdhury, S.; Paredes-Juarez, G. A.; Guix, M.; Bi, C.; Johnson, B.; English, B. W.; Jafari, S.; Baker-McKee, J.; Watson-Daniels, J. Magnetically aligned nanorods in alginate capsules (MANiACs): Soft matter tumbling robots for manipulation and drug delivery. *Micromachines* **2019**, *10* (4), 230.

(49) Bozuyuk, U.; Yasa, O.; Yasa, I. C.; Ceylan, H.; Kizilel, S.; Sitti, M. Light-triggered drug release from 3D-printed magnetic chitosan microswimmers. *ACS nano* **2018**, *12* (9), 9617-9625.

(50) Shitanda, I.; Terashima, M.; Sattayasamitsathit, S.; Hoshi, Y.; Itagaki, M. A Green and Biocompatible Magnetically Powered Nickel–Flagella Nanomotor. *Chemistry Letters* **2015**, *44* (3), 300-302.

(51) Maier, A. M.; Weig, C.; Oswald, P.; Frey, E.; Fischer, P.; Liedl, T. Magnetic propulsion of microswimmers with DNA-based flagellar bundles. *Nano letters* **2016**, *16* (2), 906-910.

(52) Li, J.; Angsantikul, P.; Liu, W.; Esteban-Fernández de Ávila, B.; Chang, X.; Sandraz, E.; Liang, Y.; Zhu, S.; Zhang, Y.; Chen, C. Biomimetic platelet-camouflaged nanorobots for binding and isolation of biological threats. *Advanced Materials* **2018**, *30* (2), 1704800.

(53) Yan, X.; Zhou, Q.; Vincent, M.; Deng, Y.; Yu, J.; Xu, J.; Xu, T.; Tang, T.; Bian, L.; Wang, Y.-X. J.; et al. Multifunctional biohybrid magnetite microrobots for imaging-guided therapy. *Science Robotics* **2017**, *2* (12), eaaq1155.

(54) Yan, X.; Xu, J.; Zhou, Q.; Jin, D.; Vong, C. I.; Feng, Q.; Ng, D. H.; Bian, L.; Zhang, L. Molecular cargo delivery using multicellular magnetic microswimmers. *Applied Materials Today* **2019**, *15*, 242-251.

(55) Wang, X.; Cai, J.; Sun, L.; Zhang, S.; Gong, D.; Li, X.; Yue, S.; Feng, L.; Zhang, D. Facile fabrication of magnetic microrobots based on spirulina templates for targeted delivery and synergistic chemo-photothermal therapy. *ACS applied materials & interfaces* **2019**, *11* (5), 4745-4756.

(56) Liu, L.; Chen, B.; Liu, K.; Gao, J.; Ye, Y.; Wang, Z.; Qin, N.; Wilson, D. A.; Tu, Y.; Peng, F. Wireless manipulation of magnetic/piezoelectric micromotors for precise neural stem-like cell stimulation. *Advanced Functional Materials* **2020**, *30* (11), 1910108.

(57) Zhang, Y.; Zhang, L.; Yang, L.; Vong, C. I.; Chan, K. F.; Wu, W. K.; Kwong, T. N.; Lo, N. W.; Ip, M.; Wong, S. H. Real-time tracking of fluorescent magnetic spore–based microrobots for remote detection of C. diff toxins. *Science advances* **2019**, *5* (1), eaau9650.

(58) Dong, R.; Wang, C.; Wang, Q.; Pei, A.; She, X.; Zhang, Y.; Cai, Y. ZnO-based microrockets with light-enhanced propulsion. *Nanoscale* **2017**, *9* (39), 15027-15032.

(59) Du, S.; Wang, H.; Zhou, C.; Wang, W.; Zhang, Z. Motor and rotor in one: Light-active ZnO/Au twinned rods of tunable motion modes. *Journal of the American Chemical Society* **2020**, *142* (5), 2213-2217.

(60) Xuan, M.; Wu, Z.; Shao, J.; Dai, L.; Si, T.; He, Q. Near Infrared Light-Powered Janus Mesoporous Silica Nanoparticle Motors. *Journal of the American Chemical Society* **2016**, *138* (20), 6492-6497.

(61) Shao, J.; Abdelghani, M.; Shen, G.; Cao, S.; Williams, D. S.; van Hest, J. C. M. Erythrocyte Membrane Modified Janus Polymeric Motors for Thrombus Therapy. *ACS Nano* **2018**, *12* (5), 4877-4885.

(62) Wu, Z.; Li, T.; Gao, W.; Xu, T.; Jurado-Sánchez, B.; Li, J.; Gao, W.; He, Q.; Zhang, L.; Wang, J. Cell-membrane-coated synthetic nanomotors for effective biodetoxification. *Advanced Functional Materials* **2015**, *25* (25), 3881-3887.

(63) Esteban-Fernández de Ávila, B.; Angsantikul, P.; Ramírez-Herrera, D. E.; Soto, F.; Teymourian, H.; Dehaini, D.; Chen, Y.; Zhang, L.; Wang, J. Hybrid biomembrane–functionalized nanorobots for concurrent removal of pathogenic bacteria and toxins. *Science Robotics* **2018**, *3* (18), eaat0485.

(64) Wu, Z.; De Avila, B. E.-F.; Martín, A.; Christianson, C.; Gao, W.; Thamphiwatana, S. K.; Escarpa, A.; He, Q.; Zhang, L.; Wang, J. RBC micromotors carrying multiple cargos towards potential theranostic applications. *Nanoscale* **2015**, *7* (32), 13680-13686.

(65) Wu, Z.; Li, J.; de Ávila, B. E. F.; Li, T.; Gao, W.; He, Q.; Zhang, L.; Wang, J. Water-powered cell-mimicking Janus micromotor. *Advanced Functional Materials* **2015**, *25* (48), 7497-7501.

(66) Mou, F.; Chen, C.; Zhong, Q.; Yin, Y.; Ma, H.; Guan, J. Autonomous motion and temperature-controlled drug delivery of Mg/Pt-poly (N-isopropylacrylamide) Janus micromotors driven by simulated body fluid and blood plasma. *ACS applied materials & interfaces* **2014**, *6* (12), 9897-9903.

(67) Li, J.; Angsantikul, P.; Liu, W.; Esteban-Fernández de Ávila, B.; Thamphiwatana, S.; Xu, M.; Sandraz, E.; Wang, X.; Delezuk, J.; Gao, W.; et al. Micromotors Spontaneously Neutralize Gastric Acid for pH-Responsive Payload Release. *Angewandte Chemie International Edition* **2017**, *56* (8), 2156-2161.

(68) de Ávila, B. E.-F.; Angsantikul, P.; Li, J.; Angel Lopez-Ramirez, M.; Ramírez-Herrera, D. E.; Thamphiwatana, S.; Chen, C.; Delezuk, J.; Samakapiruk, R.; Ramez, V. Micromotor-enabled active drug delivery for in vivo treatment of stomach infection. *Nature communications* **2017**, *8* (1), 1-9.

(69) Gao, W.; Dong, R.; Thamphiwatana, S.; Li, J.; Gao, W.; Zhang, L.; Wang, J. Artificial Micromotors in the Mouse's Stomach: A Step toward in Vivo Use of Synthetic Motors. *ACS Nano* **2015**, *9* (1), 117-123.

(70) Li, J.; Thamphiwatana, S.; Liu, W.; Esteban-Fernández de Ávila, B.; Angsantikul, P.; Sandraz, E.; Wang, J.; Xu, T.; Soto, F.; Ramez, V. Enteric micromotor can selectively position and spontaneously propel in the gastrointestinal tract. *ACS nano* **2016**, *10* (10), 9536-9542.

(71) Zhou, M.; Hou, T.; Li, J.; Yu, S.; Xu, Z.; Yin, M.; Wang, J.; Wang, X. Self-propelled and targeted drug delivery of poly (aspartic acid)/iron–zinc microrocket in the stomach. *Acs Nano* **2019**, *13* (2), 1324-1332.

(72) Sattayasamitsathit, S.; Kou, H.; Gao, W.; Thavarajah, W.; Kaufmann, K.; Zhang, L.; Wang, J. Fully-loaded micromotors for combinatorial delivery and autonomous release of cargoes. *Small (Weinheim an der Bergstrasse, Germany)* **2014**, *10* (14), 2830.

(73) Xuan, M.; Shao, J.; Lin, X.; Dai, L.; He, Q. Self-Propelled Janus Mesoporous Silica Nanomotors with Sub-100 nm Diameters for Drug Encapsulation and Delivery. *ChemPhysChem* **2014**, *15* (11), 2255-2260.

(74) Ma, X.; Jannasch, A.; Albrecht, U.-R.; Hahn, K.; Miguel-López, A.; Schaffer, E.; Sánchez, S. Enzyme-powered hollow mesoporous Janus nanomotors. *Nano letters* **2015**, *15* (10), 7043-7050.

(75) Tu, Y.; Peng, F.; André, A. A.; Men, Y.; Srinivas, M.; Wilson, D. A. Biodegradable hybrid stomatocyte nanomotors for drug delivery. *Acs Nano* **2017**, *11* (2), 1957-1963.

(76) Tu, Y.; Peng, F.; White, P. B.; Wilson, D. A. Redox-sensitive stomatocyte nanomotors: destruction and drug release in the presence of glutathione. *Angewandte Chemie International Edition* **2017**, *56* (26), 7620-7624.

(77) Toebes, B. J.; Abdelmohsen, L. K.; Wilson, D. A. Enzyme-driven biodegradable nanomotor based on tubular-shaped polymeric vesicles. *Polymer Chemistry* **2018**, *9* (23), 3190-3194.

(78) Wu, Z.; Wu, Y.; He, W.; Lin, X.; Sun, J.; He, Q. Self-propelled polymer-based multilayer nanorockets for transportation and drug release. *Angewandte Chemie International Edition* **2013**, *52* (27).

(79) Wu, Z.; Lin, X.; Zou, X.; Sun, J.; He, Q. Biodegradable protein-based rockets for drug transportation and light-triggered release. *ACS Applied Materials & Interfaces* **2015**, *7* (1), 250-255.

(80) Sugai, N.; Nakai, Y.; Morita, Y.; Komatsu, T. Transparent protein microtubule motors with controllable velocity and biodegradability. *ACS Applied Nano Materials* **2018**, *1* (7), 3080-3085.

(81) Sugai, N.; Morita, Y.; Komatsu, T. Nonbubble-propelled biodegradable microtube motors consisting only of protein. *Chemistry–An Asian Journal* **2019**, *14* (17), 2953-2957.

(82) Nakai, Y.; Sugai, N.; Kusano, H.; Morita, Y.; Komatsu, T. Protein microtube motors with a Pt nanoparticle interior and avidin exterior for self-propelled transportation, separation, and stirring. *ACS Applied Nano Materials* **2019**, *2* (8), 4891-4899.

(83) Choi, H.; Hwang, B. W.; Park, K. M.; Kim, K. S.; Hahn, S. K. Degradable nanomotors using platinum deposited complex of calcium carbonate and hyaluronate nanogels for targeted drug delivery. *Particle & Particle Systems Characterization* **2020**, *37* (1), 1900418.

(84) Kim, D.; Liu, A.; Diller, E.; Sitti, M. Chemotactic steering of bacteria propelled microbeads. *Biomedical microdevices* **2012**, *14* (6), 1009-1017.

(85) Park, S. J.; Park, S.-H.; Cho, S.; Kim, D.-M.; Lee, Y.; Ko, S. Y.; Hong, Y.; Choy, H. E.; Min, J.-J.; Park, J.-O. New paradigm for tumor theranostic methodology using bacteria-based microrobot. *Scientific reports* **2013**, *3* (1), 3394.

(86) Stanton, M. M.; Simmchen, J.; Ma, X.; Miguel-López, A.; Sánchez, S. Biohybrid Janus motors driven by Escherichia coli. *Advanced Materials Interfaces* **2016**, *3* (2), 1500505.

(87) Akin, D.; Sturgis, J.; Ragheb, K.; Sherman, D.; Burkholder, K.; Robinson, J. P.; Bhunia, A. K.; Mohammed, S.; Bashir, R. Bacteria-mediated delivery of nanoparticles and cargo into cells. *Nature Nanotechnology* **2007**, *2* (7), 441-449.

(88) Luo, C.-H.; Huang, C.-T.; Su, C.-H.; Yeh, C.-S. Bacteria-mediated hypoxia-specific delivery of nanoparticles for tumors imaging and therapy. *Nano letters* **2016**, *16* (6), 3493-3499.

(89) Cho, S.; Park, S. J.; Ko, S. Y.; Park, J.-O.; Park, S. Development of bacteria-based microrobot using biocompatible poly (ethylene glycol). *Biomedical microdevices* **2012**, *14* (6), 1019-1025.

(90) Li, D.; Choi, H.; Cho, S.; Jeong, S.; Jin, Z.; Lee, C.; Ko, S. Y.; Park, J. O.; Park, S. A hybrid actuated microrobot using an electromagnetic field and flagellated bacteria for tumor-targeting therapy. *Biotechnology and bioengineering* **2015**, *112* (8), 1623-1631.

(91) Singh, A. V.; Hosseinidoust, Z.; Park, B.-W.; Yasa, O.; Sitti, M. Microemulsion-Based Soft Bacteria-Driven Microswimmers for Active Cargo Delivery. *ACS Nano* **2017**, *11* (10), 9759-9769.

(92) Taherkhani, S.; Mohammadi, M.; Daoud, J.; Martel, S.; Tabrizian, M. Covalent binding of nanoliposomes to the surface of magnetotactic bacteria for the synthesis of self-propelled therapeutic agents. *ACS nano* **2014**, *8* (5), 5049-5060.

(93) Alapan, Y.; Yasa, O.; Schauer, O.; Giltinan, J.; Tabak, A. F.; Sourjik, V.; Sitti, M. Soft erythrocyte-based bacterial microswimmers for cargo delivery. *Science Robotics* **2018**, *3* (17), eaar4423.

(94) Weibel, D. B.; Garstecki, P.; Ryan, D.; DiLuzio, W. R.; Mayer, M.; Seto, J. E.; Whitesides, G. M. Microoxen: Microorganisms to move microscale loads. *Proceedings of the National Academy of Sciences* **2005**, *102* (34), 11963-11967.

(95) Chen, C.; Chang, X.; Angsantikul, P.; Li, J.; Esteban-Fernández de Ávila, B.; Karshalev, E.; Liu, W.; Mou, F.; He, S.; Castillo, R. Chemotactic guidance of synthetic organic/inorganic payloads functionalized sperm micromotors. *Advanced Biosystems* **2018**, *2* (1), 1700160.

(96) Karshalev, E.; Zhang, Y.; Esteban-Fernández de Ávila, B.; Beltrán-Gastélum, M.; Chen, Y.; Mundaca-Uribe, R.; Zhang, F.; Nguyen, B.; Tong, Y.; Fang, R. H. Micromotors for active delivery of minerals toward the treatment of iron deficiency anemia. *Nano letters* **2019**, *19* (11), 7816-7826.

(97) Wang, H.; Chen, X.; Qi, Y.; Wang, C.; Huang, L.; Wang, R.; Li, J.; Xu, X.; Zhou, Y.; Liu, Y.; Xue, X. Self-Propelled Nanomotors with an Alloyed Engine for Emergency Rescue of Traumatic Brain Injury. *Advanced Materials* **2022**, *34* (49), 2206779.

(98) Xie, L.; Pang, X.; Yan, X.; Dai, Q.; Lin, H.; Ye, J.; Cheng, Y.; Zhao, Q.; Ma, X.; Zhang, X. Photoacoustic imaging-trackable magnetic microswimmers for pathogenic bacterial infection treatment. *ACS nano* **2020**, *14* (3), 2880-2893.

(99) Cao, W.; Liu, Y.; Ran, P.; He, J.; Xie, S.; Weng, J.; Li, X. Ultrasound-propelled janus rod-shaped micromotors for site-specific sonodynamic thrombolysis. *ACS Applied Materials & Interfaces* **2021**, *13* (49), 58411-58421.

(100) Wan, M.; Wang, Q.; Li, X.; Xu, B.; Fang, D.; Li, T.; Yu, Y.; Fang, L.; Wang, Y.; Wang, M. Systematic research and evaluation models of nanomotors for cancer combined therapy. *Angewandte Chemie International Edition* **2020**, *59* (34), 14458-14465.

(101) Liu, K.; Huang, R.; Li, W.; Yao, J.; Lei, D.; Yang, G.; Huang, Z.; Chen, L.; Sun, H.; Xiong, Z.; et al. Geometry-Programmable Light-Driven Silicon Microrobots. *Advanced Materials* **2026**, *n/a* (n/a), e22384.

(102) Li, N.; Zhou, M.; Wu, Z.; Chen, Y.; Duan, Y.; Zhang, Z.; Wu, Z.; Xia, X.; Shen, J.; Mao, C.; Wan, M. Artificial Mitochondrial Nanorobots Deliver Energy In Vivo by Oral Administration. *Advanced Materials* **2025**, *37* (24), 2500495.
